# Oxidative cleavage of polysaccharides by a termite-derived *superoxide dismutase* boosts the degradation of biomass by glycoside hydrolases†

**DOI:** 10.1039/d1gc04519a

**Published:** 2022-05-12

**Authors:** João Paulo L. Franco Cairo, Fernanda Mandelli, Robson Tramontina, David Cannella, Alessandro Paradisi, Luisa Ciano, Marcel R. Ferreira, Marcelo V. Liberato, Lívia B. Brenelli, Thiago A. Gonçalves, Gisele N. Rodrigues, Thabata M. Alvarez, Luciana S. Mofatto, Marcelo F. Carazzolle, José G. C. Pradella, Adriana F. Paes Leme, Ana M. Costa-Leonardo, Mário Oliveira-Neto, André Damasio, Gideon J. Davies, Claus Felby, Paul H. Walton, Fabio M. Squina

**Affiliations:** Department of Biochemistry and Tissue Biology, Institute of Biology, University of Campinas (UNICAMP) Campinas São Paulo Brazil; Department of Geosciences and Natural Resource Management, Faculty of Science, University of Copenhagen Rolighedsvej 23 DK-1958 Frederiksberg C Denmark; Department of Chemistry, University of York York YO10 5DD UK paul.walton@york.ac.uk; Brazilian Biorenewables National Laboratory, Brazilian Center for Research in Energy and Materials Campinas São Paulo Brazil; Programa de Processos Tecnológicos e Ambientais da Universidade de Sorocaba (UNISO) Sorocaba SP Brazil fabio.squina@prof.uniso.br; Departamento de Física e Biofísica, Instituto de Biociências, Universidade Estadual Paulista, UNESP Botucatu São Paulo Brasil; Programa de Mestrado e Doutorado em Biotecnologia Industrial, Universidade Positivo Curitiba PR Brasil; Laboratório de Genômica e Expressão, Departamento de Genética, Evolução e Bioagentes, Instituto de Biologia, Universidade de Campinas, UNICAMP Campinas São Paulo Brasil; Laboratório Nacional de Biociências (LNBio) do Centro Nacional de Pesquisa em Energia e Materiais (CNPEM) Campinas São Paulo Brasil; Laboratório de Cupins, Departamento de Biologia Geral e Aplicada, Instituto de Biociências, Universidade Estadual Paulista, UNESP Rio Claro São Paulo Brasil

## Abstract

Wood-feeding termites effectively degrade plant biomass through enzymatic degradation. Despite their high efficiencies, however, individual glycoside hydrolases isolated from termites and their symbionts exhibit anomalously low effectiveness in lignocellulose degradation, suggesting hereto unknown enzymatic activities in their digestome. Herein, we demonstrate that an ancient redox-active enzyme encoded by the lower termite *Coptotermes gestroi*, a Cu/Zn superoxide dismutase (*Cg*SOD-1), plays a previously unknown role in plant biomass degradation. We show that *Cg*SOD-1 transcripts and peptides are up-regulated in response to an increased level of lignocellulose recalcitrance and that *Cg*SOD-1 localizes in the lumen of the fore- and midguts of *C. gestroi* together with termite main cellulase, *Cg*EG-1-GH9. *Cg*SOD-1 boosts the saccharification of polysaccharides by *Cg*EG-1-GH9. We show that the boosting effect of C*g*SOD-1 involves an oxidative mechanism of action in which *Cg*SOD-1 generates reactive oxygen species that subsequently cleave the polysaccharide. SOD-type enzymes constitute a new addition to the growing family of oxidases, ones which are up-regulated when exposed to recalcitrant polysaccharides, and that are used by Nature for biomass degradation.

## Abstract 2

A Cu/Zn superoxide dismutase isolated from termite gut can oxidise glucose polymers, boosting the activity of glycoside hydrolases.

## Graphical abstract

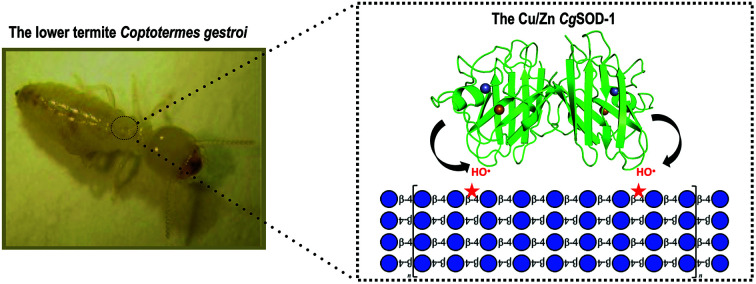

## 1. Introduction

Lignocellulosic biomass (LB) is the main feedstock in the production of second-generation biofuels.^1^ Derived from the plant cell walls (PCWs), the carbohydrate portion of LB is composed mainly of the following polysaccharides: cellulose, (40–50% of the biomass total composition), diverse hemicelluloses (25–30%), and pectin (5–10%). Lignin comprises around 20–25% of LB depending on origin.^2^ The complex 3D structures of the biopolymers in LB hinder its use as a feedstock in biofuel production, since its sustainable breakdown into soluble saccharides is both chemically and mechanically difficult. As such, LB demands extensive pre-treatment steps to reduce both its physical and chemical recalcitrance before it can be processed into soluble sugars by enzymes.^2^ At the operational level of a biorefinery, this degradation is currently achieved through the addition of enzyme cocktails which contain a combination of glycoside hydrolases (GHs) and accessory enzymes such as carbohydrate esterases. This enzymatic saccharification of the plant material is a crucial, yet expensive, processing step during the production of second-generation biofuels.^3^

Biomass-degrading organisms have evolved a variety of enzymes to overcome LB recalcitrance. However, despite the effectiveness of these organisms in utilizing LB, this efficiency is not yet fully replicated by industrial cocktails.^4^ Therefore, in the last decade, studies have concentrated on discovering what additional carbohydrate-active enzymes (CAZymes) can increase LB degradation efficiency when used in conjunction with other enzymes. A significant recent breakthrough in this regard was the discovery of Cu-containing lytic polysaccharide monooxygenases (LPMOs)^5,6^ belonging to the Auxiliary Activities (AAs) families AA9-AA11, AA13-AA17.^7^ These enzymes catalyze the oxidative cleavage of various polysaccharides (cellulose, hemicelluloses, pectin, starch and chitin), boosting significantly the activity of the enzymatic cocktails during biomass degradation both in Nature and industry.^7^ Also, as part of the endeavor to discover new enzymes, the exploration of Nature's biodiversity throughout ‘omics’ studies, has now been extended beyond bacteria and fungi,^8–10^ to look at more diverse organisms, for instance firebrats, beetles and termites.^11–13^

In this context, termites, particularly within the worker caste, are notably efficient decomposers of LB, degrading up to 90% of the ingested plant biomass.^14^ In what might be coined ‘the perfect biorefinery’ studies have shown how termites first reduce particle size in their mandibles before arrival of the masticated LB in the fore and midguts where endogenous cellulases, glucosidases and other CAZymes, such as esterases and laccases, liberate monosaccharides and oligosaccharides.^15^ Finally, the part-digested material moves to the hindgut where CAZymes from symbiotic microorganisms generate sugars that will be fermented to acetate and short-chain fatty acids that are the primary source of termite nutrition.^16,17^

Given their high efficiency, termites now present an opportunity for the discovery of new enzymes that could be used in biomass utilization.^11,18^ In this regard, many studies have focused on the lower termite *Coptotermes gestroi*,^19–21^ which was introduced in Brazil at the beginning of the last century and rapidly became a major urban pest.^22^ The especially high biomass-degrading capacity of *C. gestroi* has made it a rich source of enzymes, with some already used in biomass-to-bioproducts applications.^23,24^ Notwithstanding its potential however, studies on the use of *C. gestroi* and other termite-derived glycoside hydrolases, from both endogenous and symbiotic origins, have repeatedly and somewhat puzzlingly shown that the catalytic efficiencies of isolated enzymes on recalcitrant PCW are low.^20,25–27^ This observation shows that our understanding of the consortia of enzymes used by these organisms to degrade biomass is incomplete. Our current study thus seeks to determine the ‘missing’ enzymatic components (auxiliary activity, AA enzymes) in the digestive secretome of *C. gestroi*.

In terms of what is currently understood about AA enzymes in termites, it was recently in shown that two LPMOs belonging to the family AA15 are chitin-active enzymes in *C. gestroi*, but that these have no ostensible roles in lignocellulose degradation.^28^*C. gestroi* and other lower termites do secrete in their guts non-LPMO redox-active enzymes related to pro-oxidation, antioxidation and detoxification processes (dubbed PAD enzymes).^18,19,27^ These PADs include p450s, alcohol dehydrogenases, glutathione *S*-transferases, catalases, aldo-keto reductases (AKR) and copper-dependent superoxide dismutases (SOD).^19,29,30^ Yet, despite their prevalence, there is limited direct evidence about the role of PAD enzymes in lignocellulose degradation and detoxification of lignin-derived phenolic compounds in termite gut or other organisms.^27,31^

Among these potential new families of AA enzymes, those classified as Cu/Zn superoxide dismutases perhaps are the most promising since previous studies have shown that the SOD group displays strikingly high expression levels in termites that had been fed with lignocellulose^19,27^ or in bacteria and fungi grown on plant biomass.^8,29,32^ Indeed, a patent claims that Cu/Zn SOD from termite can increase the saccharification of lignocelluloses when mixed with termite endogenous and symbiotic GHs.^33^

SOD enzymes are widely distributed. They catalyze the dismutation of the superoxide anion radical (O_2_˙^−^) into oxygen and hydrogen peroxide.^34^ SODs are metalloenzymes, divided into four families depending on the metals found at the active site: Cu/Zn, Ni, Mn, or Fe.^34^ One of the most studied is the Cu/Zn SOD group, carrying in their catalytic site a copper ion (Cu^2+^) and a zinc ion (Zn^2+^) linked by an imidazolate bridge. The redox-active Cu directly interacts with the substrates in the following pair of reactions:

1 Cu^2+^-SOD + O_2_˙^−^ → Cu^+^-SOD + O_2_

2 Cu^+^-SOD + O_2_˙^−^ + 2H^+^ → Cu^2+^-SOD + H_2_O_2_

Beyond their principal role of superoxide scavenging, SODs are also known to produce hydroxyl radicals (˙OH) when exposed to high concentrations of hydrogen peroxide.^35–37^ For the purposes of the study described herein, the ˙OH radical is able to cleave glucose-based polysaccharides, generating several oxidized ion products as recently reported by Boulos and Nyström.^38^ Fenton chemistry (Fe^2+^ + H_2_O_2_ → Fe^3+^ + ˙OH + OH^−^) is similarly utilized by brown-rot fungi to depolymerize lignocellulosic biomass.^39^

Here we report the isolation of a Cu/Zn SOD from *C. gestroi*. We show that during termite feeding experiments on sugarcane bagasse, *Cg*SOD-1 transcripts and peptides are up-regulated in response to the increased level of lignocellulose recalcitrance. We further demonstrate that *Cg*SOD-1 localizes in the fore- and midgut of *C. gestroi* together with its major cellulase. Furthermore, this SOD works in synergy with endogenous termite GHs for polysaccharide breakdown, although this boosting effect is reduced/disappears when other biomass-degrading enzymes are added, revealing the complex relationship between enzyme components and the efficiency of biomass degradation. We show that this SOD degrades polysaccharides through an oxidative mode of action, from which it boosts the action of glycosidic hydrolases in an analogous manner to that of LPMO enzymes. Thus *Cg*SOD-1 adds a new member to the group of ‘Auxiliary Activity’ enzymes used by Nature in biomass utilization.

## 2. Results

### Lignocellulose recalcitrance influences Cu/Zn SOD expression in *C. gestroi*

**Fig. 1:**
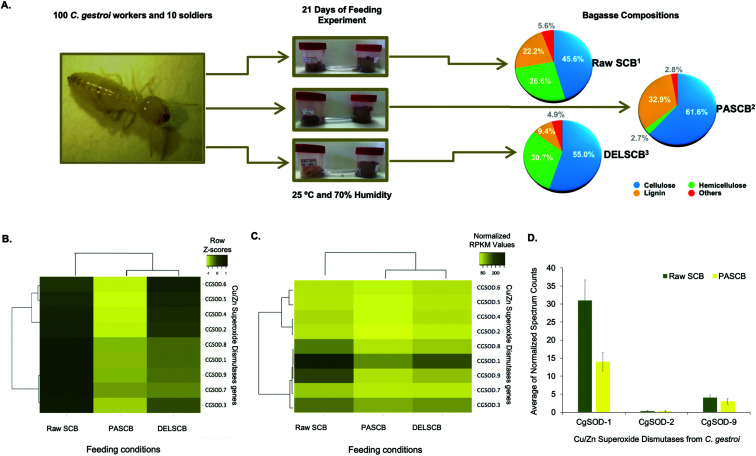
Feeding assay scheme and plots from the differential expression of superoxide dismutase in workers of *C. gestroi* fed on different sugarcane bagasse. (A) Schematic view of feeding assays using 100 *C. gestroi* workers and 10 soldiers. ^(1)^ The compositional analysis of Raw Sugarcane Bagasse (SCB) was: 45.6% cellulose, 26.6% hemicellulose, and 22.2% lignin.^40^^(2)^ Pre-treated Phosphoric Acid Sugarcane Bagasse (PASCB) has 61.7% cellulose, 2.8% hemicellulose, and 33.0% lignin^41^ and ^(3)^ Delignified Sugarcane Bagasse (DELSCB) has 55% cellulose, 30.7% hemicellulose, and 9.4% lignin.^42^ (B) Nine Cu/Zn SOD genes were identified previously in the genome of *C. gestroi.* Heat map analysis of the *Z*-score for the complete-linkage hierarchical clustering based on Pearson distance measure, showing the differential gene expression pattern of Cu/Zn SODs in Raw SCB, PASCB and DELSCB diets. (C) Heat map analysis showing the number of normalized RPKMs for each diet. The normalized RPKM was represented by the average of RPKMs among the replicates. Colour from light yellow to dark yellow indicates low to high gene transcription levels. (D) The normalized peptide spectrum counts for three endogenous Cu/Zn SOD enzymes from *C. gestroi* between raw SCB and PASCB feeding conditions.

Recently, the expression of PAD enzymes was reported to be up-regulated when termites were fed on lignocelluloses.^18,19,27^ The expression of nine putative genes encoding for Cu/Zn SODs, identified in the genome of *C. gestroi*,^19^ was evaluated by transcriptomics and proteomics after 21 days of a feeding experiment. Termite workers were fed either raw sugarcane bagasse (SCB), pretreated phosphoric acid sugarcane bagasse (PASCB) or pretreated sodium chlorite/HCl delignified sugarcane bagasse (DELSCB) as sole carbon sources (Fig. 1a). The highest abundance of SOD transcripts was observed in SCB-fed animals, as shown in the heat map chart (Fig. 1b). According to the dendrogram analysis (Fig. 1c), the gene expression of *CGSOD-1*, *CGSOD-8* and *CGSOD-9* clustered together, and these transcripts presented the highest number of normalized Reads Per Kilobase Millions (RPKMs) among all Cu/Zn SODs. The same three genes presented a near doubling in up-regulation in SCB compared to both PASCB and DELSCB (log_2_ of fold change <−0.5 and False Discovery Rate (FDR) <0.05) (Table S1†). Similarly, the expression of two main *C. gestroi*'s cellulases from family GH9 (*CGEG1* and *CGEG2*) as well as the two main β-glucosidases GH1 (*CGBG1* and *CGBG2*) was also monitored, revealing that these enzymes are also highly up-regulated in response to SCB compared to the other feeding conditions (Table S1†).

Following on from the transcriptomics, proteomics similarly identified three Cu/Zn SODs (Fig. 1d), including two differentially expressed in the RNA-Seq: *Cg*SOD-1 and *Cg*SOD-9. The proteome revealed that *Cg*SOD-1 was differentially expressed, resulting in levels ∼2× higher in SCB-fed termites than PASCB-fed individuals (*p*-value: 0.0032). *Cg*SOD-1 also exhibited the highest number of spectrum counts (and total peptides), among the three Cu/Zn SODs identified (Table S2†). The two major *C. gestroi* cellulases (*Cg*EG-1 and *Cg*EG-2) and β-glucosidases (*Cg*BG-1 and *Cg*BG-2) were likewise identified in the proteomics analysis. Collectively, our data support that *Cg*SOD-1 and cellulases are overproduced in SCB-feeding experiments compared to other conditions (Table S2†).

Since SCB was not pretreated, it represented the material with the highest recalcitrance level (*i.e.*, lower porosity and higher crosslinking between the polysaccharides and lignin).^40^ PASCB has 90% less hemicellulose than SCB as well as partially modified lignin, as a result of the pre-treatment process.^41^ Likewise, DELSCB presents instead a lower lignin content (about 65% less) than SCB and a comparable amount of hemicellulose, albeit the latter is modified as a result of the pre-treatment step.^42^ Thus, the up-regulation of SOD transcript and proteins during feeding, especially for *Cg*SOD-1, correlates with the degree of LB in this substrate.

### *CGSOD-1* encodes a canonical Cu/Zn superoxide dismutase

**Fig. 2:**
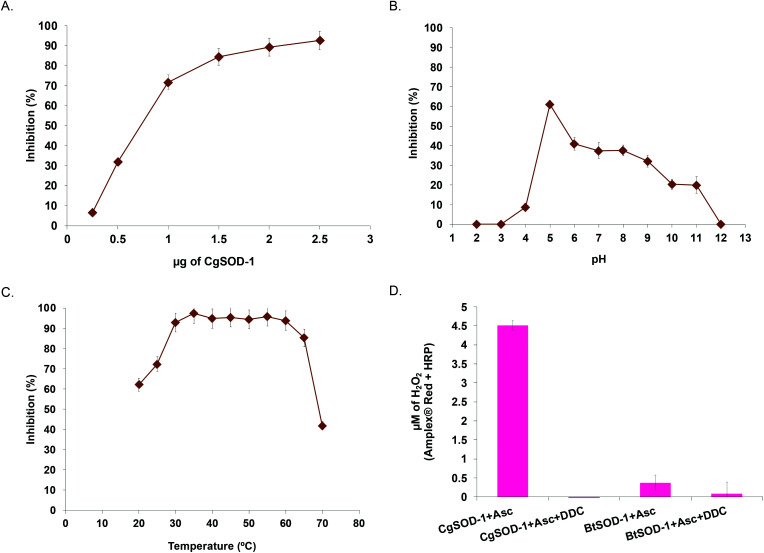
Biochemical characterization of *Cg*SOD-1. (A) Inhibition percentage of pyrogallol autoxidation at different *Cg*SOD-1 concentrations. Inhibition percentage of pyrogallol autoxidation by 2.5 μg of *Cg*SOD-1 at different pHs (B) and different temperatures (C). Generation of hydrogen peroxide by *Cg*SOD-1 and *Bt*SOD-1 (D) in the absence or presence of inhibitor DDC measured by Amplex® Red method (50 μM Amplex + 7 U mL^−1^ of HRP final concentrations). All the biochemical assays were done in triplicate.

To evaluate whether *CGSOD-1* encodes a functional superoxide dismutase, the gene was amplified from a *C. gestroi* cDNA library and heterologously expressed in *E. coli* (Fig. S1a†). *Cg*SOD-1 production was confirmed by LC-MS/MS (Fig. S1B and C†) and its initial activity was assayed using the pyrogallol method (inhibition of autoxidation), presenting an IC_50_ of 1.17 μg (4.7 μg mL^−1^) (Fig. 2a). This is a high scavenger activity when compared with bacterial (1.6 mg mL^−1^)^43^ and fungal (1.3 mg mL^−1^)^44^ SODs. In addition, *Cg*SOD-1 was active for superoxide dismutation from pH 4.0 to 11.0, with the optimum activity around pH 5.0 (Fig. 2b). The optimum temperature activity ranged from 30 °C to 60 °C, retaining ∼80% of the activity from 30 °C to 65 °C, (Fig. 2c and S2a,† respectively). The *Cg*SOD-1 secondary folding (Fig. S2b†) and thermal stability were confirmed by Far-UV Circular Dichroism (CD), giving a melting temperature of 66(1) °C (*R*^2^ = 0.9996) (Fig. S2c†). *Cg*SOD-1 produced around 4.5 μmol L^−1^ of H_2_O_2_ (after 30 min reaction at 30 °C), using ascorbic acid as electron/superoxide donor (Fig. 2d) and activity was lost in the presence of the Cu chelator diethyldithiocarbamate (DDC). *Cg*SOD-1 displayed higher activity than the prototypical bovine Cu/Zn *Bt*SOD-1 from *Bos taurus*, (Fig. 2d).

**Fig. 3:**
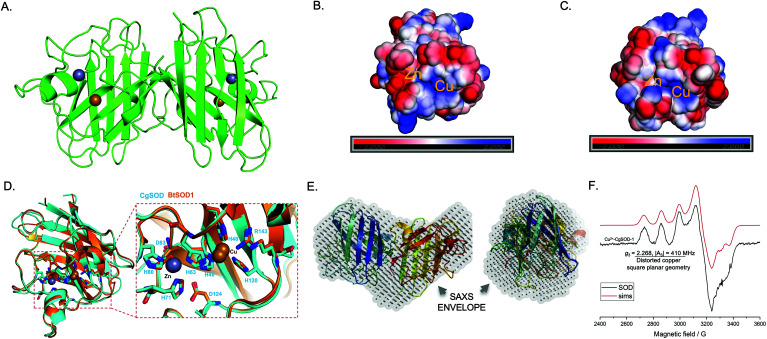
Structural and spectroscopic hallmarks for *Cg*SOD-1. (A) Cartoon representation of the homology model for *Cg*SOD-1 as a homodimer, showing the Zn (grey sphere) and Cu (golden sphere) metals in the catalytic pocket. The model was generated by SWISS-MODEL server, using the crystal structure PDB: 3L9Y from *Bombyx mori* as a template. The electrostatic potential surface at pH 6.0 of *Cg*SOD-1 (B) and *Bt*SOD-1 (C) shows the positively charged region (blue) present on both protein surfaces around the Cu ion. Around the Zn ion, a neutral (white) and positively charged surface (blue) is shown for *Bt*SOD-1 and negatively charged (red) for *Cg*SOD-1. (D) The active site of *Cg*SOD-1 (blue) superposed with equivalent residues from the commercial Cu/Zn-SOD *Bt*SOD-1 (orange) (1E9Q pdb) from *Bos Taurus*. All the residues that have contact with the metals are conserved in *Cg*SOD-1. (E) SAXS analysis showing the superposition of ‘*ab initio*’ dummy atom model (DAM) (in grey) for *Cg*SOD-1 envelope and the crystal structure *BmCu-Zn*SOD (3L9Y). The left model is rotated 90° along the *x*-axis from the right model. (F) Continuous-wave X-band EPR spectrum (black line) at 9.3 GHz and 160 K with simulation (red line) for *Cg*SOD-1. The spectrum shows a typical type 2 copper (Cu^2+^) at the active site for *Cg*SOD-1.

Given the high sequence similarity of *Cg*SOD-1 to previous Cu/Zn SODs (around 75%), a three-dimensional (3D) structure of *Cg*SOD-1 was generated using SWISS-MODEL (QMEAN of 1.08) and the crystal structure PDB: 3L9Y from *Bombyx mori* as a template. The *Cg*SOD-1 was assembled as a homodimer (subunits A and B) in the 3D model, showing a typical β-barrel folding, with eight β-sheets and several loops. The copper and zinc metals ions were also modelled in the active site (Fig. 3a), according to their analogous positions in other Cu/Zn SODs. The electrostatic surface potentials for *Cg*SOD-1 and *Bt*SOD-1 were also calculated and, as for other SODs, a positively charged pocket of amino acids was found around the Cu ion for both proteins (Fig. 3b and c). Conversely, *Cg*SOD-1 has an overall negative net charge around the Zn ion, while *Bt*SOD-1 is positively and neutrally charged in the same region. A detailed comparison between the active site of *Cg*SOD-1 and *Bt*SOD-1 (PDB: 1E9Q) was performed, which confirmed highly conserved positions of amino acids around the metals (Fig. 3D), including the copper-His63-zinc imidazolate bridge essential for the enzymatic mechanism of Cu/Zn SODs. According to small-angle X-ray scattering (SAXS) experiments (Fig. S3a–d†), *Cg*SOD-1 shows high flexibility with an estimated molecular mass in solution of 33.2 kDa, indicating the predominance of homodimers. The SAXS ‘*ab initio’* model was also built and the three-dimensional dummy atom model (DAM) of *Cg*SOD-1 was determined from the SAXS curves (Fig. 3e) (Table S3†), confirming the homodimer configuration as well as validating the molecular modelling prediction.

X-band Electron Paramagnetic Resonance (EPR) spectroscopy studies were conducted to determine the electronic state of the active site copper from *Cg*SOD-1. The frozen solution spectrum of the protein (Fig. 3f) is consistent with what was previously reported in the literature for other Cu/Zn SODs.^45,46^ The spin Hamiltonian parameters derived from the simulations of the continuous wave (cw) EPR spectrum of *Cg*SOD-1 are indicative of a type 2 copper site according to the Peisach-Blumberg classification,^47^ with a certain degree of distortion from square planar geometry (*g*_3_ = 2.268, |*A*_3_| = 410 MHz) (Table S4†). The overall spectral envelope is characterized by a SOMO with mostly d(*x*^2^ − *y*^2^) character with small amounts of d(*z*^2^) mixing, that would arise from a distorted square planar structure.

### *Cg*SOD-1 is co-located with the termite main endo-cellulase and the site of H_2_O_2_ production *in vivo*

**Fig. 4:**
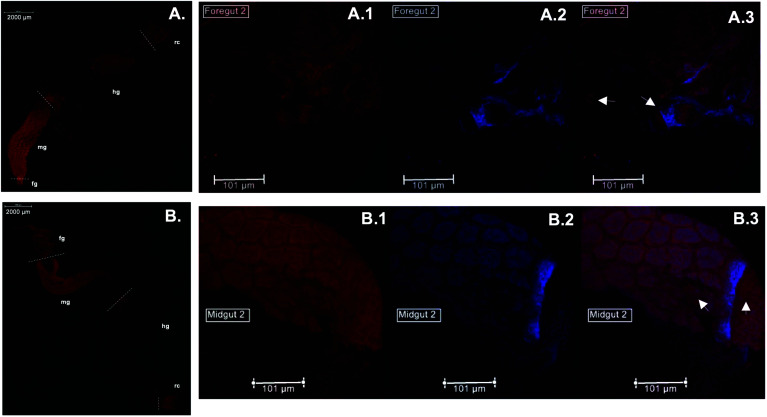
Immunolocalization of *Cg*EG-1 and *Cg*SOD-1 in *C. gestroi* gut tissues. Gut tissues were incubated with primary anti-*Cg*EG-1 (A) or anti-*Cg*SOD-1 (B) antibodies and fluorescent secondary antibodies (AlexaFluoor 568 fluorophores). The images were recorded using a stereomicroscope with Epi-Fluorescence Illuminator (Nikon's SMZ1500) with TRITC filter (red). The foregut of *C. gestroi* with primary anti-*Cg*EG-1 and secondary antibody in red (A.1), with Prolong DAPI blue (A.2) and the superimpose of both images (A.3). The midgut of *C. gestroi* with primary anti-*Cg*SOD-1 and secondary antibody in red (B.1), with Prolong DAPI blue (B.2) and the superimpose of both images (B.3). Legends (fg – foregut, mg – midgut, hg – hindgut and rc – rectum). The images are the best representation of biological triplicates. The slides were mounted using ProLong™ Antifade Reagents with or without DAPI for fixed cells and the close images were observed on a Leica DMI 6000 microscope.

Immunolocalization assays using anti-*Cg*SOD-1 and anti-*Cg*EG-1 (endo-β-1,4-glucosidase 1 from *Coptotermes gestroi*) antibodies were performed on dissected *C. gestroi* guts. Here, the localization of *Cg*EG-1 can be considered as a positive control because it is expected to locate in the foregut and midgut regions^48^ (Fig. 4a). *Cg*EG-1 was found in the lumen of the gut region (Fig. 4 a.1 and a.3, controls in Fig. S4a, b and c†). For *Cg*SOD-1, the gut tissue similarly shows a strong signal in the midgut, as well as in the foregut, mainly in the pro-ventricle part. A low fluorescence signal was also observed for *Cg*SOD-1 in the rectum, as well as around the enteric valve (Fig. 4b), whilst no signal was detected at the hindgut. The expanded view from the midgut segment shows that *Cg*SOD-1 is outside of the gut cells, confirming its presence in the lumen of the midgut (Fig. 4 b.1 and b.3).

**Fig. 5:**
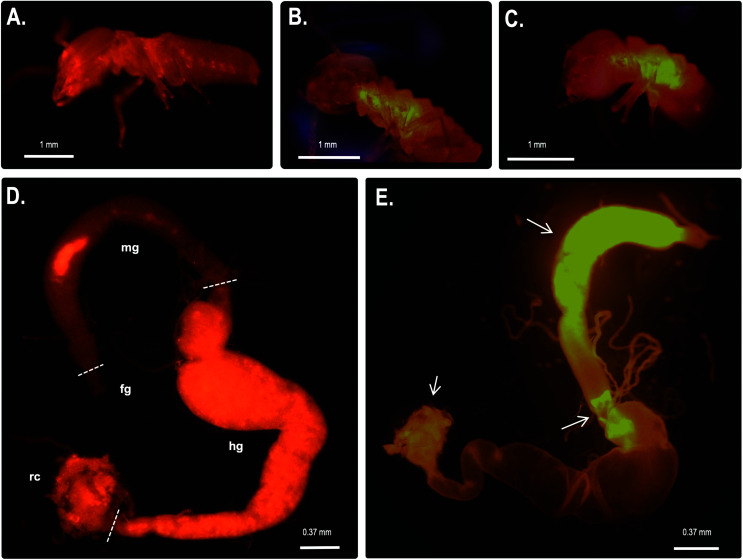
*In vivo* localization of hydrogen peroxide generation in *C. gestroi* guts. Workers of *C. gestroi* were incubated with Amplex Red/HRP work solution and observed under fluorescence microscope: (A) – control before incubation; (B) – ventral and (C) – lateral views after Amplex Red/HRP incubation. A strong fluorescence signal in the midgut is detected. The dissected gut tissues of *C*. *gestroi* workers were also incubated with Amplex Red/HRP work solution. (D) – control (fg – foregut, mg – midgut, mt – Malpighian tubes, g – hindgut and rc – rectum); (E) – gut tissues after incubation with Amplex Red. A strong fluorescence signal in the midgut is evident. The low fluorescence signal was verified at the beginning of the hindgut and anus region (see the arrows). The images were recorded using a stereomicroscope with Epi-Fluorescence Illuminator (Nikon's SMZ1500) with TRITC filter (red). The images are the best representation of biological triplicates.

We also performed fluorescence microscopy to evaluate H_2_O_2_ production *in vivo* using the Amplex® Red reagent. As shown in Fig. 5b and c, a strong signal was detected in the thorax and abdomen of *C. gestroi*, demonstrating the formation of hydrogen peroxide inside the gut, further confirmed after gut dissection and Amplex® Red labelling. The strong *Cg*SOD-1 immunofluorescence, in the fore- and midguts therefore correlates strongly with H_2_O_2_ production *in vivo*, Fig. 5f.

### *Cg*SOD-1 boosts the activity of termite glycoside hydrolases, promoting substrate oxidation through the generation of reactive oxygen species

Previous reports from our group and the literature have shown that the cellulase *Cg*EG-1-GH9 works in synergy for the saccharification of β-glucan and carboxymethylcellulose (CMC) with the main termite β-glucosidase, *Cg*BG-1-GH1, and also with another PAD enzyme, the aldo-keto reductase 1 from *C. gestroi* (*Cg*AKR-1).^20,27,31^*Cg*SOD-1 localizes in the fore- and mid-guts of *C. gestroi* together with *Cg*EG-1-GH9, and therefore we investigated whether *Cg*SOD-1 could similarly boost the activity of *Cg*EG-1 on model substrates. The degree of synergism (DS) among the enzymes was calculated as *abc*/(*a* + *b* + *c*) as described previously,^49^ where *abc* is the result in μmol of sugar released by the enzymes together and *a*, *b* and *c* are the results of each enzyme alone. DS ≥ 1.1 indicates a synergism effect for the combination of the enzymes.

#### Boosting of the action of single enzymes by CgSOD-1

**Fig. 6:**
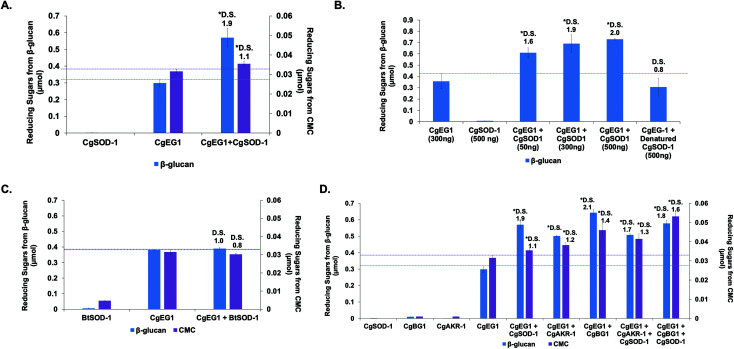
Synergy assays of *Cg*SOD-1 with enzymes from *C. gestroi*. The reactions were conducted at 30 °C for 30 min, using 0.25% (w/v) β-glucan or CMC as substrates in 50 mM sodium acetate buffer pH 6.0. (A) Synergism assays between *Cg*EG-1 and *Cg*SOD-1 for β-glucan (purple) or CMC (blue) saccharifications. (B) Synergism assays between *Cg*EG-1 and different amounts of *Cg*SOD-1. A total of 300 ng of *Cg*EG-1 was used in all assays, while different amounts of *Cg*SOD-1 were tested (50, 300 and 500 ng). Denatured *Cg*SOD-1 was used as a negative control. (C) Synergism assays between *Cg*EG-1 and *Bt*SOD-1 for β-glucan (purple) or CMC (blue) saccharifications. A total of 300 ng of *Cg*EG-1 as well as for *Bt*SOD-1 were used. (D) Synergism assays among *Cg*EG-1, *Cg*SOD-1, *Cg*AKR-1 and *Cg*BG-1. A total of 100 ng of each enzyme was used in the experiments. After all the assays, the total reducing sugars were measured with the 3,5-dinitrosalicylic acid (DNS) method. The degree of synergism (DS) among the enzymes were calculated as μmol of equivalent glucose of *abc*/(*a* + *b* + *c*). DS ≥ 1.1 indicates a synergism effect for the combination of the enzymes. The error bars are relative to the standard error of biological triplicates.

Activity assays with β-glucan from barley (mixed β-1,3:1,4) and CMC were performed with *Cg*EG-1 in combination with *Cg*SOD-1, showing around 90% increase (DS of 1.9) of the reducing sugars released from β-glucan saccharification (0.55 μmol) and 10% from CMC (DS of 1.1–0.035 μmol), when compared to *Cg*EG-1 alone (0.30 μmol from β-glucan and 0.030 μmol from CMC) (Fig. 6a). A lower saccharification yield was expected for CMC since it is not the optimal substrate of *Cg*EG-1 (Fig. 6a).

Furthermore, the increase of *Cg*SOD-1 loads in the assays increased the reducing sugar amount released from β-glucan (Fig. 6b) from around 0.6 μmol to around 0.75 μmol, as well as the DS (1.6 to 2.0). As a control, the addition of denatured *Cg*SOD-1 did not increase the saccharification yield of *Cg*EG-1 (Fig. 6b), showing no DS as well as any putative synergistic effect of the free copper and zinc. Moreover, the ability of a different SOD, *Bt*SOD-1, to work in synergy with *Cg*EG-1 was also tested using β-glucan and CMC as substrates; however, no DS was found (Fig. 6c) in the tested conditions, and an inhibitory effect was found for CMC saccharification (DS of 0.8).

#### Boosting of the action of multiple enzymes by CgSOD-1

Additionally, *Cg*SOD-1 was combined with *Cg*EG1 in the presence of *Cg*AKR-1 or *Cg*BG-1 (Fig. 6d). For β-glucan saccharification, the addition of *Cg*SOD-1 to mixtures of either *Cg*EG-1 + *Cg*AKR-1 (DS of 1.7) or *Cg*EG-1 + *Cg*BG-1 (DS of 1.8) resulted in DS values similar to the results without *Cg*SOD-1 (DS of 1.7 for *Cg*EG-1 + *Cg*AKR-1 and 2.1 for *Cg*EG-1 + *Cg*BG-1), and with the same saccharification yields for *Cg*EG-1 + *Cg*AKR-1 and *Cg*EG-1 + *Cg*AKR-1 + *Cg*SOD-1 (0.50 μmol) or with a slightly lower saccharification yield for *Cg*EG-1 + *Cg*BG-1 + *Cg*SOD-1 (0.58 μmol) compared with *Cg*EG-1 + *Cg*BG-1 (0.65 μmol) but, without an inhibitory effect when compared to *Cg*EG-1 alone (0.30 μmol for β-glucan) (Fig. 6d).

For CMC saccharification, the presence of *Cg*SOD-1 together with *Cg*EG-1 + *Cg*AKR-1 (DS of 1.3) or *Cg*EG-1 + *Cg*BG-1 (DS of 1.6) increased the final sugar yields (0.042 and 0.055 μmol of reducing sugars respectively), when compared with the coupled enzymes (0.038 μmol for *Cg*EG-1 + *Cg*AKR-1 and 0.035 for *Cg*EG-1 + *Cg*BG-1) (Fig. 6d). Again, no inhibitory effect was found when compared with *Cg*EG-1 alone (0.03 μmol for CMC).

Interestingly, our data point to a complex picture of the synergistic effects of biomass-degrading enzymes. In the first instance, *Cg*SOD-1 significantly boosts the action of a single glycoside hydrolase. Somewhat counterintuitively however, no boosting and possibly even a slight inhibitory effect is seen when a third enzyme (*Cg*BG-1 or *Cg*AKR-1) is then added to the biomass-degrading system. While the ostensible contradiction brings into question whether *Cg*SOD-1 is a natural boosting enzyme at all, we note that the effects of enzyme degradation by oxidative chemistry and the potential for inhibition of *Cg*BG-1 by oxidized saccharides are factors which may be more evident in our laboratory-based system as compared to natural systems. For instance, both the temporal and relative concentrations of enzyme components are carefully controlled by organisms during biomass degradation—a feature absent in our current laboratory study. This constitutes an area for future study.

#### Boosting mechanism

**Fig. 7:**
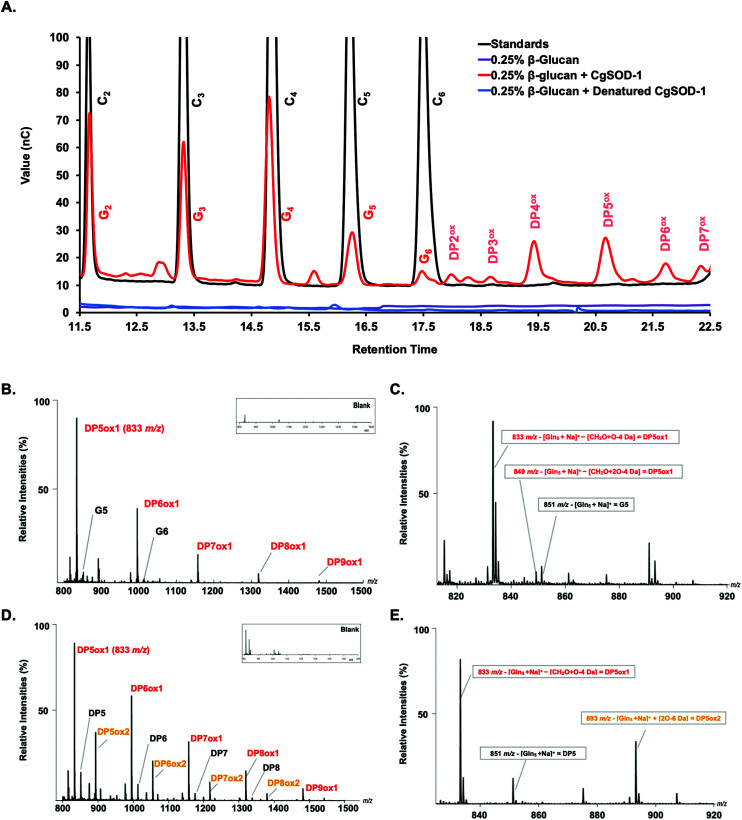
The oxidative cleavage of polysaccharides by *Cg*SOD-1. (A) HPAEC (ICS3000) chromatograms of the glucose oligosaccharides released from 0.25% β-glucan after the incubation with *Cg*SOD-1. DPn^ox^ is referred to as putative oxidation at the reducing end of the oligosaccharide of different degrees of polymerization. G2–G6 stands for native glucose oligosaccharides released from β-glucan. C2–C6 were used as cello-oligosaccharides standards. Maldi-TOF (UltraFlex III – Bruker) analysis of products released from incubation of glucose-based polymers with *Cg*SOD-1 at pH 6 and 30 °C. (B) Ion products obtained from β-glucan after 24 hours of reaction with *Cg*SOD-1 showing native and oxidized products. The main peak series identified was relative to −18 Da (DP_*n*_ox1) from its mono-sodiated non-oxidized native *gluco*-oligosaccharide (DP_*n*_ + Na^+^), with a difference of 162 Da or a hexose unit among them, suggesting a degree of polymerization (DP) from DP5 to DP9. Small box: blank reaction containing only β-glucan. (C) Expanded spectrum from DP5 showing the ion series from oxidized G5 formed after the transformation of reducing end glucose unit into arabinonic acid. (D) Ion products obtained from Avicel after 24 hours of reaction with *Cg*SOD-1 showing native and oxidized products. The main peak series identified was also relative to −18 Da (DP_*n*_ox1) from its mono-sodiated non-oxidized native oligosaccharide (DP_*n*_ + Na^+^), with a difference of 162 Da or a hexose unit among them, which suggest a DP ranging from DP5 to DP9. The second main peak series was relative to +42 Da (DP_*n*_ox2), ranging from DP5 to DP8 from its mono-sodiated non-oxidized native oligosaccharide and with a hexose unit of difference among the products. Small box: blank reaction containing only Avicel. (E) Expanded spectrum from DP5 showing the ion series from oxidized cellopentaose formed after the transformation of reducing end glucose unit into arabinonic acid (DP_5_ox1) and the ion series from oxidized cellopentaose formed after the transformation of reducing end glucose unit into gluconic acid (DP_5_ox2). All the ion products found for both substrates were compatible with ions derived from the oxidation of glucose-based polysaccharides by hydroxyl radicals generated by Fenton chemistry.

To further understand this boosting effect of *Cg*SOD-1 in the saccharification of polysaccharides by glycosidic hydrolases, *Cg*SOD-1 was incubated with β-glucan, and the products of the reaction were monitored by HPAEC-PAD chromatography.^51,52^ After two hours of incubation at 30 °C pH 6.0, it was observed that *Cg*SOD-1 released a series of native glucose oligosaccharides from β-glucan (G2 to G6) (Fig. 7a). Under the HPAEC conditions used (*i.e.*, optimised to separate oxidised from non-oxidised oligosaccharides), mixed linkage species such (β-1,3/β-1,4) overlap and could not be separated from cello-oligosaccharides (β-1,4).

Notably, peaks were also detected in the region of the chromatogram where oxidized oligosaccharides elute with a degree of polymerization (DP) ranging from 2 to 7, including four minor signals at 18, 18.5, 21.5, and 22.5 minutes of retention and two major ones at 19.5 and 20.5 minutes (Fig. 7a). Peaks with similar retention times have previously been assigned to oxidized cello-oligosaccharide with DP2 and DP3 for the early peaks (18 and 18.5 min), and to DP4, DP5, DP6 and DP7 for the later peaks (19.5, 20.5, 21.5 and 22.5 min) (Fig. 7a).^53^

In this context, we note that the native glucose oligosaccharides eluted from the reaction could be also the result of on-column C4 oxidized sugar degradation due to the high pH of the chromatography method (known for C4 oxidized oligos from substrates oxidized by LPMOs, where the C4 oligos tend to be degraded to the native sugars one unit shorter).^52^ Incubation of denatured *Cg*SOD-1 or 0.1 mmol L^−1^ CuCl_2_ with β-glucan and no signals arising from the polysaccharide degradation were detected (Fig. 7a and S5a†). Bovine Cu/Zn *Bt*SOD-1 was also incubated following the reaction conditions described above; however, again the production of putative native or oxidized oligosaccharides was not observed (Fig. S5a†).

In further confirmation of the oxidative action of *Cg*SOD-1, the degradation of β-glucan only by Celluclast® in the absence of *Cg*SOD-1 was also investigated. The HPAEC-PAD chromatogram showed that glucose oligosaccharides released by Celluclast from β-glucan contained a mixture of β-1,4 and β-1,3 linkages ranging from G2 to G6, eluting in similar retention time (Fig. S5b†) to cello-oligosaccharides (only β-1,4) as previously reported.^54,55^ Interestingly, no oxidative products were identified from these reactions.

To validate further the enzyme-dependent production of oxidized sugars, β-glucan or Avicel were incubated with *Cg*SOD-1 for 24 h and the reaction products analyzed by MALDI/ToF MS. Interestingly, the series of ion products, showing both native and oxidized gluco-oligosaccharides (Table 1), were comparable to previous reports of gluco-oligosaccharide oxidation through Fenton chemistry.^38,56^ For β-glucan, the acidic product ions were detected at *m*/*z* 833, 995, I1157, 1319 and 1481 (DP_*n*_ox1), indicating an ion series with DP ranging from DP5 to DP9 (Glc_5_ox1 to Glc_9_ox1), separated by 162 Da, a hexose molecule (Fig. 7b). According to Simões *et al.*^56^ and Boulos and Nyström,^38^ the loss of 18 Da from sodiated non-oxidized native species ([Glc_*n*_ + Na]^+^) is equivalent to a loss of a [CH_2_O + O − 4 Da] (Fig. 7c) from the reducing end glucose unit, which corresponds to the formation of arabinonic acid product due to oxidative cleavage of the C1–C2 bond combined with the formation of two keto groups along the oligosaccharide chain (Table 1).

**Table 1:** List of oxidation products identified by MALDI-TOF-MS

Products	Mass difference^a^	n5	n6	n7	n8	n9
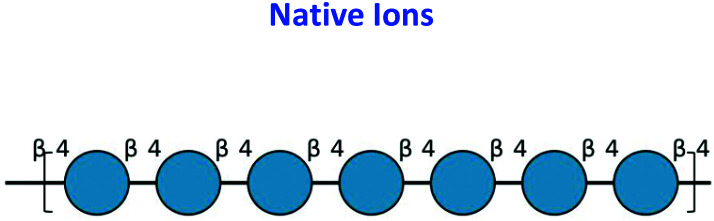						
Glc_*n*_ + Na^+^		*851*	*1013*	*1175*	*1337*	—
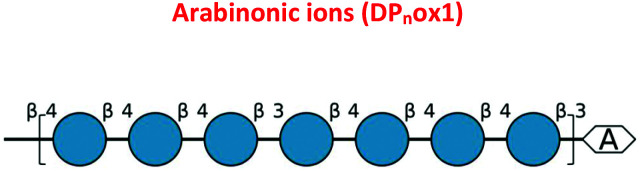						
Glc_*n*_ + Na^+^ − (CH_2_O + O-4 Da)	(−18)	*833*	*995*	*1157*	*1319*	*1481*
Glc_*n*_ + Na^+^ − (CH_2_O + 2O-4 Da)	(−2)	*849*	*1011*	—	—	—
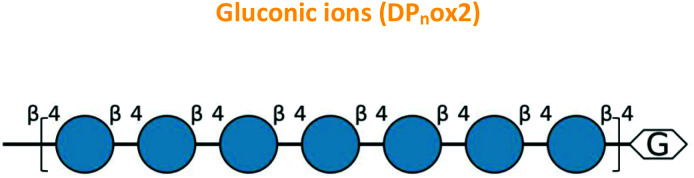						
Glc_*n*_ + Na^+^ + (3O-6 Da) ox2	(+42)	*893*	*1055*	*1217*	*1379*	—

a From the sodiated native product (Glc_*n*_ + Na^+^).

Another ion product from the arabinonic series with a loss of −2 Da was also identified at *m*/*z* 849 (Glc_5_ –CH_2_O + 2O − 4 Da) (Fig. 7c) (Table 1). Similar to the ion products derived from β-glucan oxidation (DP_*n*_ox1), the Avicel degradation also exhibited another series of ion products at *m*/*z* 893, 1055, 1217, and 1379 (DP_*n*_ox2) (Fig. 7d), which corresponds to a gain of 42 Da to [Glc_*n*_ + Na]^+^ (Fig. 7e) (Table 1). These ion products were also separated by a hexose monomer (162 Da), suggesting an ion series with a degree of polymerization ranging from DP5 to DP8. The gain of 42 Da from the native ion is equivalent to the generation of gluconic acid (Glc_*n*_ + 3O − 6 Da) in the reducing end glucose unit with a formation of three keto groups along the oligosaccharide (Fig. 7e) (Table 1).

#### Generation of reactive oxygen species by CgSOD-1

**Fig. 8:**
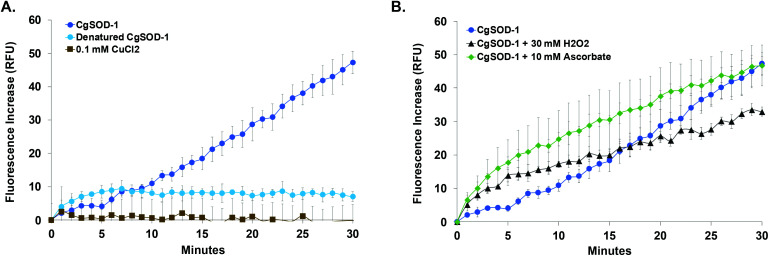
The generation of hydroxyl radicals by *Cg*SOD-1. Fluorescence increasing relative to the hydroxyl radical (˙OH) formation detected by HPF probe at pH 6. (A) Reaction with 50 ng of *Cg*SOD-1, 50 ng of denatured *Cg*SOD-1 (10 min boiled and 10 min frost) and 0.1 mM of CuCl_2_. Denatured *Cg*SOD-1 and 0.1 mM of CuCl_2_ were not to generate fluorescence. (B). Reaction with 50 ng of *Cg*SOD-1 in the presence of 30 mM hydrogen peroxide (used to induce the production of hydroxyl radicals by Cu/Zn SODs) and 10 mM ascorbic acid (used as superoxide anion donor to Cu/Zn SODs).

Previous reports have suggested that, in specific conditions, Cu/Zn SODs and MnSODs release hydroxyl radicals (˙OH).^36,37^ Therefore, the ability of *Cg*SOD-1 to generate ˙OH was investigated and monitored kinetically (Fig. 8) using 2-6-(4-hydroxy) phenoxy-3*H*-xanthan-3-on-9-yl-benzoic acid (HPF) as a fluorescent probe.^57^ In accordance with the hypothesis described herein, *Cg*SOD-1 was indeed able to produce ˙OH after 30 minutes of reaction. Denatured *Cg*SOD-1 or 0.1 mmol L^−1^ CuCl_2_ could not generate hydroxyl radicals (Fig. 8a), showing that the production of ˙OH is *Cg*SOD-1 dependent (denatured enzyme controls in Fig. S5c†).

Furthermore, reactions in the presence of 10 mmol L^−1^ ascorbic acid or 30 mmol L^−1^ hydrogen peroxide (in these concentrations, the ascorbic acid can act as a reducing agent/superoxide donor for SODs^58^ and hydrogen peroxide can stimulate the production of hydroxyl radicals by Cu/Zn SODs or MnSODs^35–37^) revealed that *Cg*SOD-1, in the presence of both compounds, could initially increase the velocity of hydroxyl radical generation faster than *Cg*SOD-1 alone (Fig. 8b). However, after 30 minutes of reaction, *Cg*SOD-1 alone achieved equivalent or higher amounts of ˙OH (Fig. 8b), suggesting that the presence of these compounds at the tested concentrations and incubation time, may cause damage and inactivation of *Cg*SOD-1.

### *Cg*SOD-1 increases the saccharification of sugarcane bagasse by commercial and laboratory fungal CAZymes preparations

**Fig. 9:**
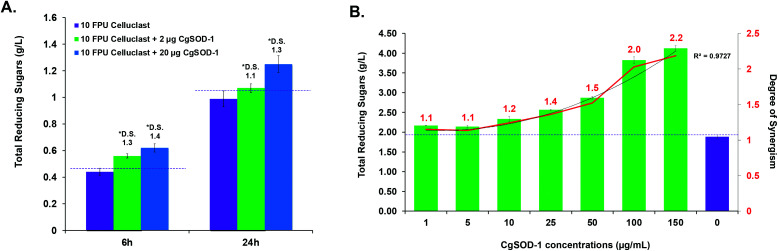
Synergistic effects of *Cg*SOD-1 added in Celluclast® and *P. echinulatum* secretome for saccharification of pretreated sugarcane bagasse. (A) Supplementation assays were performed with 10 FPU of ®Celluclast and two different loads of *Cg*SOD-1 (2 and 20 μg). The saccharification was performed at 30 °C and mixed at 1000 rpm for 24 hours, with 2% (w/v) of steam-exploded sugarcane bagasse (BEX) as substrate, in 1.5 mL of total reaction volume. After the assays, the total reducing sugars were measured with the 3,5-dinitrosalicylic acid (DNS) method and the DS was calculated. DS ≥ 1.1 indicates a synergism effect for the combination of the enzymes. The highest DS found was 1.26 after 24 hours of saccharification using 10 FPU of Celluclast® and 20 μg of *Cg*SOD-1. Among all the experiments, this condition released the highest amount of reducing sugar. (B) 0.33 FPU of *P. echinulatum* secretome was mixed with 5% (w/v) of hydrothermally pretreated sugarcane bagasse as the substrate in 100 mM phosphate buffer pH 4.8 a 50 °C. Different amounts of *Cg*SOD-1 were supplemented in the reactions (0–150 μg mL^−1^) to reach a final volume of 1.5 mL and mixed at 50 °C, 1000 rpm for 24 hours using a thermo mixer (ThermoMixer C, Eppendorf). The degree of synergism (DS) between the *Cg*SOD-1 and the cocktails were calculated described above.

The ability of *Cg*SOD-1 to boost recalcitrant plant biomass saccharification by fungal lignocellulolytic CAZymes was also assessed. Two different Celluclast® loadings (5 and 10 FPU) and two different concentrations of *Cg*SOD-1 were used on steam-exploded pretreated sugarcane bagasse (Fig. 9a and S6a†). After 6 hours of reaction, the assays containing 10 FPU Celluclast® exhibited DS of 1.3 and 1.4 for 2 and 20 μg of *Cg*SOD1, respectively (Fig. 9a). After 24 hours, the reactions with 20 μg CgSOD-1 exhibited a DS of 1.3, reaching the highest reducing sugar release (1.2 g L^−1^) (Fig. 9a and Fig S6b†). Notably, and consistent with previous data, no synergistic effects were detected in the assays containing ®Celluclast with 20 μg of *Bt*SOD-1 (Fig. S6c†). Similarly, DDC, a specific inhibitor for Cu/Zn SODs prevented the synergistic action of *Cg*SOD-1 (Fig. S6d†).

Supplementation of non-commercial *Penicillium echinulatum* hypercelluloytic secretome (free of preservatives) similarly exhibited an increase of saccharification yields of hydrothermally pretreated sugarcane bagasse in response to the *Cg*SOD1 (Fig. 9b), reaching around 4 g L^−1^ of reducing sugar. In addition, the degree of synergy using 50 μg mL^−1^ of *Cg*SOD-1 was 1.5, with 100 μg mL^−1^ enzyme was 2.0 and with 150 μg mL^−1^ enzyme was 2.2, indicating that the increase of *Cg*SOD-1 concentration in the reaction increased the degree of synergism (*R*^2^ = 0.9727) and the sugar yields.

## 3. Discussion

In the last decade, reports on termite digestome have revealed the occurrence of CAZy and PAD enzymes^18,19,27^ but biochemical assays with classical cellulases from termites (GH9),^20,26^ as well as from their protist symbionts (GH5, GH7 and GH45),^27,50^ have demonstrated anomalously poor saccharification efficiencies. These findings led to the hypothesis described herein of whether auxiliary mechanisms, such as oxidative enzymes, are also involved in the termite digestome,^18,19,31^ through the production of ROS and subsequent oxidative cleavage of polysaccharide chains.^31,32,37,59–61^ In this context, PAD genes and enzymes are found at high concentrations in the worker caste of *C. gestroi*, commensurate with their roles in feeding the colony, rather than colony defense.^19^ Among these PAD enzymes, a highly expressed enzyme that annotates as a superoxide dismutase is of note, not least because SODs have previously been reported to be involved in lignin oxidation and production of Fenton reaction intermediates.^36,37^

Our results show that the global expression of endogenous Cu/Zn SODs genes is upregulated in response to LB feeding. The plant biomass with the highest degree of recalcitrance tested in our experiments is consistent with previous reports on PAD gene upregulation in the lower termites *Reticulitermes flavipes.*^27^ and *Coptotermes formosanus*.^62^ We further show that *Cg*SOD-1 and H_2_O_2_ production localise to the midgut and foregut of *C. gestroi*, a microoxic environment with an average pH of 6.0. This phenomenon is similar to that reported in the wood-feeding lower termites *R. flavipes*,^63^ and *Coptotermes formosanus*^64^ as well as for the litter-feeding *Cornitermes cumulans*, which also exhibit high levels of ROS in the fore- and mid-guts, allied with a high abundance of mRNA for *Cc*SD (Cu/Zn SOD) and glutathione peroxidase (*Cc*GPX).^65–67^

*Cg*SOD-1 mediates an oxidative mechanism for the degradation of glucose polymers akin to that of the product profile exhibited by fungal and bacterial LPMOs^5,6,68,69^ and likewise acts in synergy with classical glycoside hydrolases. Notably, activity on β-glucan and CMC reported here is specific for *Cg*SOD-1, no effects were observed for *Bt*SOD-1, one of the most studied vertebrate Cu/Zn SODs.^70,71^ This model SOD has similar biochemical properties to *Cg*SOD-1, such as optimal pH around 5.5–6 and optimal temperature 25–30 °C.^70^

Furthermore, although *Bt*SOD-1 and *Cg*SOD-1 share the same amino acids in the coordination sphere of Cu and Zn, there are several different residues around the active site. The electrostatic potential in both enzymes’ catalytic pockets was very similar around the copper metal (both positively charged). However, *Cg*SOD-1 exhibits more negatively charged residues around the zinc metal, *i.e.*, the substitution K68D. The shift from a positively charged protonated amino acid to a negatively charged deprotonated residue may explain the catalytic differences between these enzymes.

## 4. Conclusions

For the last two decades, scientists have reported that detritivore insect guts, such as caterpillars, beetles, and termites, produce considerable amounts of reactive oxygen species (ROS) involved in the degradation of plant-derived phenolic compounds and tannins.^13,65,66^ Recent studies have hypothesized that this ROS generation could enhance plant cell wall degradation by boosting the activity of endogenous cellulases.^19,27,31^ It is in this regard that the role Cu/Zn SODs discovered in our report now solves a long-running puzzle of how detritivore insect guts produce ROS in their guts and why endogenous termite hydrolases do not exhibit high catalytic performance on insoluble and recalcitrant polysaccharides. As such, *Cg*SOD-1 can now be seen as an essential component of *C. gestroi* digestion physiology, working in synergy with other GH enzymes for plant biomass saccharification through the generation of redox species.

## Author contributions

JPLFC performed and/or conceived: RNA-Seq and proteomics experiments, cloning and protein expressions/purifications; enzymatic activities and saccharification experiments, conceived the immunolocalizations experiment and analyzed all data. FM performed protein expressions and purifications as well as enzymatic activities. RT performed saccharification assays and immunolocalization microscopies. DC conceived and performed the HPAEC experiments. AP performed Maldi-TOF MS experiments. LC performed EPR experiments and simulations. MRF collected SAXS data. MVL performed structural molecular modelling. LBB and TAG performed the HPAEC and HPLC experiments and performed saccharification assays. GNR and JGCP performed fungal cultivation, and fungal saccharification experiments. TMA performed protein expressions and purifications for SAXS analyses. LSM and MCF performed bioinformatic analysis. AFPL conceived the proteomics experiments. AMCL conceived termite feeding assays and provide the termites. MON conceived the SAXS experiments and analyzed the data. AD conceived cloning-expression and protein purifications as well as enzymatic activities. CF conceived the HPAEC analysis and analyzed the data. PHW conceived the EPR experiment and analyzed the data. FMS conceived and coordinated all the experiments. JPLFC, FM and DC drafted the manuscript with contributions from all authors. JPLFC, CF, GJD, PHW and FMS wrote and revised the paper.

## Conflicts of interest

The authors declare competing financial interests: Several of the authors have submitted patent (BR 10 2015 017256 7 A2) on the use of Cu/Zn SODs for biomass degradation and valorization.

## Supplementary Material

GC-024-D1GC04519A-s001
