# Supplementary material for: Oxidative cleavage of polysaccharides by a termite-derived superoxide dismutase boosts the degradation of biomass by glycoside hydrolases
Source: Green Chem. 2022 May 12;24(12):4845–58. doi: 10.1039/d1gc04519a (PMC9208272; doi:10.1039/d1gc04519a)
Supplement: GC-024-D1GC04519A-s001 [file GC-024-D1GC04519A-s001.pdf]

Electronic Supplementary Material (ESI) for Green Chemistry.
This journal is © The Royal Society of Chemistry 2022

Supporting Information for

Oxidative cleavage of polysaccharides by a termite-derived *superoxide dismutase* boosts the degradation of biomass by glycoside hydrolases

João Paulo L. Franco Cairo,[a,b,c] Fernanda Mandelli,[d] Robson Tramontina,[e] David Cannella,[b,†] Alessandro Paradisi,[c] Luisa Ciano,[c,#] Marcel R. Ferreira,[f] Marcelo V. Liberato,[e] Lívia B Brenelli,[b,d] Thiago A. Gonçalves,[a] Gisele N. Rodrigues,[d] Thabata M. Alvarez,[g] Luciana S. Mofatto,[h] Marcelo F. Carazzolle,[h] José G. C. Pradella,[d] Adriana F. Paes Leme,[i] Ana M. Costa-Leonardo,[j] Mário Oliveira-Neto,[f] André Damasio,[a] Gideon J. Davies,[c] Claus Felby,[b] Paul H. Walton,[c,*] Fabio. M. Squina,[e,*]

Author Addresses

[a]Department of Biochemistry and Tissue Biology, Institute of Biology, University of Campinas (UNICAMP), Campinas, São Paulo, Brazil.

[b]Department of Geosciences and Natural Resource Management, Faculty of Science, University of Copenhagen, Rolighedsvej 23, DK-1958 Frederiksberg C, Denmark.

[c]Department of Chemistry, University of York, York, YO10 5DD, United Kingdom.

[d]Brazilian Biorenewables National Laboratory, Brazilian Center for Research in Energy and Materials, Campinas, São Paulo, Brazil

[e]Programa de Processos Tecnológicos da Universidade de Sorocaba (UNISO), Sorocaba, SP, Brazil.

[f]Departamento de Física e Biofísica, Instituto de Biociências, Universidade Estadual Paulista, UNESP, Botucatu, São Paulo, Brasil.

[g]Programa de Mestrado e Doutorado em Biotecnologia Industrial, Universidade Positivo, Curitiba, PR, Brasil.

[h]Laboratório de Genômica e Expressão, Departamento de Genética, Evolução e Bioagentes, Instituto de Biologia, Universidade de Campinas, UNICAMP, Campinas, São Paulo, Brasil.

[i]Laboratório Nacional de Biociências (LNBio) do Centro Nacional de Pesquisa em Energia e Materiais (CNPEM), Campinas, São Paulo, Brasil.

[j]Laboratório de Cupins, Departamento de Biologia, Instituto de Biociências, Universidade Estadual Paulista, UNESP, Rio Claro, São Paulo, Brasil.

Corresponding Authors

[*]paul.walton@york.ac.uk and fabio.squina@prof.uniso.br

Present Addresses

[†]PhotoBioCatalysis unit, Crop Production and Biostimulation, Biomass Transformation lab, Université Libre de Bruxelles, Campus Plaine CP245, 1050-Brussels, Belgium.

[#]School of Chemistry, University of Nottingham, University Park, Nottingham, NG7 2RD, United Kingdom.

This PDF file includes:

Experimental

Supporting Results

Supporting Figures. S1 to S6

Supporting Tables S1 to S4

Experimental

Termites and feeding experiments

Specimens of *C. gestroi* (Wasmanm, 1986) were maintained in the Termite Laboratory of the Biology Department, UNESP, Rio Claro, São Paulo, Brazil (22° 23'S, 47° 31'W) after collection from field colonies using traps of corrugated cardboard. Termites were kept at 25 ± 2 °C and fed on corrugated cardboard with 10% of moisture until feeding experiments. The feeding experiments were performed in arenas with slight modifications from what has been previously described.[1] These arenas were composed of a central release chamber (145 mL) connected by small plastic tubes (10 cm long and 0.5 cm in diameter) to the food chamber (145 mL). The release chamber contained a moistened matrix of sterile sand (80% moisture content), which completely covered the insertion hole of the connecting tube. One hundred workers and 10 soldiers of *C. gestroi* were placed in the central chamber, and the termites could enter the food chambers immediately following their introduction. As food, three different lignocellulosic materials were used: raw Sugarcane Bagasse (SCB), Pre-treated Phosphoric Acid Sugarcane Bagasse (PASCB) and Delignified Sugarcane Bagasse (DELSCB). One gram of substrate was placed in each food chamber. Five replicates were established for each diet, and all experiments were maintained at 25 ± 1°C for 21 days. Afterwards, 100 workers were collected from the food chamber, frozen with liquid nitrogen and kept at -80 °C until RNA or protein extractions. The substrates were kindly provided by the industrial division of the Brazilian Bioethanol Science and Technology Laboratory – CTBE (Campinas, SP, Brazil) and the University of São Paulo - USP (Lorena, SP, Brazil). The reported compositional analysis of the materials is: 45.6% cellulose, 26.6% hemicellulose, and 22.2% lignin for SCB,[2] 61.6% cellulose, 2.7% hemicellulose, and 32.9% lignin for PASCB,[3] and 55% cellulose, 30.7% hemicellulose, and 9.4 % lignin for DELSCB.[4]

Total RNA extraction, rRNA depletion and RNA-Seq library preparation and sequencing

Total RNA (10 µg) was extracted from 50 workers of each biological replicate from the feeding experiment using Trizol reagent protocol (Invitrogen). The Trizol/Chloroform step was performed twice. The total RNA was purified using the RNeasy Plant Mini Kit (Qiagen) following the manufacturer's instructions. The quality of RNA was verified using RNAnano chip Bioanalyzer 2100 (Agilent). Good quality RNAs (RIN > 8.0) were submitted to rRNA depletion using the RiboZero rRNA Removal Kit under manufacturer instruction with a slight modification: at the depletion step, we used a blend of rRNA removal solution from RiboZero Gold and RiboZero Bacteria kits (1:1) aiming to deplete both prokaryotic and eukaryotic rRNAs. Finally, the depleted RNAs were purified using Ampure XP beads, following the manufacturer's instructions, and kept at -80 °C until RNAseq library preparation. A total of 50 ng of depleted RNA for each replicate from the feeding experiment were used for library preparation using Stranded TruSeq RNA Sample Preparation kit, following the manufacturer's instructions. The protocol began at the fragmentation and prime step from the Stranded TruSeq RNA guide. Quality control and quantification of the libraries were performed using a DNA 1000 series II Bioanalyzer Chip (Agilent) and KAPA Library Quantification Kit for NGS, respectively. For each library, paired-end sequences of 100 bp were generated in a single lane using Illumina HiSeq 2500, from the High Throughput Sequencing and Robotics Laboratory at CTBE (Campinas, Brazil). A total of 9 libraries were generated from workers fed on Raw SCB, DELSBC and PASCB (3 each) and the raw data sequence was deposited at NCBI under the Bioproject PRJNA335415.

Determination of Gene Expression Levels

The SOD, cellulases and actin genes were previously identified and annotated in the genome of *C. gestroi.*[5] The differential gene expression was measured via the Reads Per Kilobase Million (RPKM) method, using only one read alignment for each transcript[6] for each feeding condition and the prediction of signal peptide was performed in WoLF PSORT platform.[7] The heatmap graph was generated, using Complete-Linkage as clusterization method and Pearson as a distance measurement method for rows and columns. Log-fold change and false discovery rate (FDR) values were obtained using the EdgeR R/Bioconductor package.[6] As a control, the global analysis of seven constitutive genes coding for actin (Pfam00022) did not reveal any significant differences among the feeding conditions (Table S1). We expected no difference between conditions for this gene since they are related to basal and structural metabolism in *C. gestroi.*

Protein extraction and mass spectrometry-based proteomic analysis

The proteins were extracted from the whole bodies of 20 workers of *C. gestroi* in biological triplicates from the RAW SCB and PASCB feeding assays after 21 days as described previously. The worker protein extract (75 µg) in triplicates from each condition was loaded into a 10% SDS-PAGE gel and bands at 10 kDa, 25 kDa, 35 kDa, 50 kDa, 80 kDa and above 80 kDa were cut, reduced with 5 mmol L^{-1} dithiothreitol (25 min at 56 °C), and then alkylated (14 mmol L^{-1} iodoacetamide, 30 min at room temperature in the dark), followed by digestion with trypsin (Promega) overnight at 37 °C. After drying in a vacuum concentrator, the samples were reconstituted using 50 µL of 0.1% formic acid to extract the peptides from the gel. The supernatant was transferred to new tubes and 4.5 µL of the resulting peptide mixture were analyzed on an ETD-enabled LTQ Velos Orbitrap mass spectrometer (Thermo Fisher Scientific) coupled with LC-MS/MS by an EASY-nLC system (Proxeon Biosystems) through a Proxeon nanoelectrospray ion source.

The peptides were separated by a 2-80% acetonitrile gradient in 0.1% formic acid using a PicoFrit Column analytical column (20 cm x ID75 µm, 5 µm particle size, new objective) at a flow rate of 300 nL min^{-1} over 27 min. The nanoelectrospray voltage was set to 2.5 kV, and the

Comment [GD]: Do we NEED that control statement here ?

Comment [JPLFC]: I moved to the SI.

source temperature was 200 °C. All instrument methods for the LTQ Velos Orbitrap were set up in the data-dependent acquisition mode. The full scan MS spectra (*m/z* 300-1,600) were acquired in the Orbitrap analyzer after accumulation to a target value of $1e^6$. The resolution in the Orbitrap was set to r = 60,000, and the 20 most intense peptide ions with charge state ≥ +2 were sequentially isolated to a target value of 5,000 and fragmented in the linear ion trap by low-energy collision-induced dissociation - CID (normalized collision energy of 35%). The signal threshold for triggering a MS/MS event was set to 1,000 counts. Dynamic exclusion was enabled with an exclusion size list of 500, exclusion duration of 60 s, and repeat count of 1. An activation q of 0.25 and an activation time of 10 ms were used.

The spectra were acquired using the software MassLynx v.4.1 (Waters - Milford, MA, USA), and the raw data files were converted to a peak list format (mgf) without summing the scans using the Mascot Distiller v.2.3.2.0 software (Matrix Science Ltd.). These spectra were searched against the *C. gestroi* database (181,554 unigenes; 42,520,001 residues - generated by the unigenes identified in the metatranscriptomic analysis described previously,[5] using the Mascot v.2.3.01 engine (Matrix Science Ltd.) with carbamidomethylation as the fixed modification, oxidation of methionine as a variable modification, one trypsin missed cleavage and a tolerance of 10 ppm for precursor ions and 1 Da for fragment ions.

All datasets processed using the workflow feature in the Mascot software were further analyzed in the software ScaffoldQ+ to validate the MS/MS-based peptide and protein identifications. Peptide identifications were accepted if they could be established at greater than 90.0% probability as specified by the Peptide Prophet algorithm.[8] Peptide identifications were also required to exceed specific database search engine thresholds. Mascot identifications required at least both the associated identity scores and ion scores to be greater than 31. Protein identifications were accepted if they could be established at greater than 80.0% probability for peptide identification. Protein probabilities were assigned using the Protein Prophet algorithm.[8] Proteins that contained similar peptides and could not be differentiated based on the MS/MS analysis alone were grouped to satisfy the principles of parsimony. The scoring parameter (Peptide Probability) in the ScaffoldQ+ software was set to obtain an FDR of less than 3%. Using the number of total spectra output from the ScaffoldQ+ software, we identified the differentially expressed proteins using spectral counting. A normalization criterion, the “quantitative value”, was applied to normalize the spectral counts and the fold change was calculated to each protein followed by Fischer’s Exact Test.

Cloning of *CGSOD-1*

The gene sequence of *CGSOD-1* (Ref Seq ALM23457) coding the full-length superoxide dismutase *Cg*SOD-1 was amplified from *C. gestroi*’s cDNA by a standard PCR method, using two primers (forward 5’- TATAGCTAGCATGCCGATAAAAGCTGTATGTGTTC-3’; reverse 5’-TATAGGATCCTTAGATCTTAGCAATTCCCACCAC-3’ - underlined sequence indicates the recognition site of the restriction enzymes *Nhe*I and *Bam*HI, respectively). The PCR product was digested with the proper enzymes followed by ligation into pET28a expression vector (Novagen), followed by the transformation in *Escherichia coli* ArcticExpress Competent Cells (Agilent) for protein expression. Physical and chemical parameters were predicted by the ProtParam tool from ExPASy (http://web.expasy.org/protparam/),[9] while the prediction of signal peptide was performed in WoLF PSORT platform [7] and secretion prediction using DeepLock 1.0.

Heterologous gene expression, protein production and purification

Cells from a single colony *of Escherichia coli* ArcticExpress DE3 Expression Cells transformed with the construction pET28a-*Cg*SOD-1 were grown in liquid LB-Miller medium supplemented with kanamycin (35 µg mL^{-1}) for 16 h at 37 °C and 200 rpm. Thereafter, the culture was inoculated in 800 mL of fresh LB medium followed by incubation at 30 °C and 200 rpm during 4 h. Next, the temperature and rotation were then reduced to 12 °C and 120 rpm, respectively. After one hour of acclimation, $CuCl_2$ and $ZnCl_2$ were added to a final concentration of 2.5 mmol L^{-1}, plus the addition of 1 mmol L^{-1} isopropyl β-D-1-thiogalactopyranoside (IPTG) to induce the gene expression and the recombinant protein production. After 24 h, the cells were harvested by centrifugation at 8,500 x*g*.

The cell pellets were resuspended in lysis buffer (20 mmol L^{-1} sodium phosphate buffer pH 7.5, 500 mmol L^{-1} NaCl, 5 mmol L^{-1} imidazole, 0.2 mg of egg lysozyme mL^{-1}, 0.02 mg mL^{-1} DNAseI and 5 mmol L^{-1} phenylmethylsulfonyl fluoride and disrupted in an ice bath by an ultrasonic processor (7 pulses of 10 s at 500 W; VC750 Ultrasonic Processor, Sonics Vibracell). Following centrifugation at 8,500 x*g*, the SOD protein present in the supernatant was purified by chromatography employing an AKTA FPLC system (GE Healthcare, Waukesha, WI, USA), with a 5 mL HisTrap HP column (GE Healthcare) charged with Ni^{2+}. Afterwards, the protein was further purified by gel filtration using a Superdex 200 10/300 GL column (GE Healthcare). The His-tag was removed using 10 U of thrombin per mg of protein after overnight digestion in the cold room followed by a new round of size-exclusion chromatography. *Cg*SOD-1 identity was confirmed by LC-MS/MS at the Mass Spectrometry Laboratory from the Brazilian Biosciences National Laboratory (LNBio), as previously reported,[10] with a FDR of 1.35%.[11] The concentration of purified *Cg*SOD-1 was measured by NanoDrop 2000c (Thermo Scientific, USA), using the molar extinction coefficient (1615 $molar^{-1}$ cm^{-1}) and compared with the Bradford method. For most of the experiments, the His6x-tag was preserved in the recombinant form of *Cg*SOD-1, having shown that its presence did not affect the results of the study (Fig. S5b).

Determination of superoxide dismutase activity

Superoxide dismutase activity was determined by a modified procedure of pyrogallol autoxidation.[12] Different amounts of the enzyme solution (0.25 to 2.5 µg) were added in a 50 mmol L^{-1} Tris-HCL containing 1 mmol L^{-1} EDTA, at pH 8.2. The reaction was performed in a microplate with 250 µL final volume and was initiated by the addition of 0.2 mmol L^{-1} pyrogallol (final concentration). The change of absorbance at 325 nm was measured every 30 s for 10 min at 25 °C. The results were expressed as the amount of enzyme in g required for 50% inhibition of pyrogallol autoxidation (IC_{50}). As control, denatured *Cg*SOD-1 was used. To evaluate the effect of pH and temperature, *Cg*SOD-1 (2.5 µg) was incubated at different pH values (pH 4.0 – 11.0) at 25 °C and temperatures (20 – 70 °C) at pH 6 before performing the pyrogallol assay. The enzyme activity under different temperatures was also evaluated by riboflavin-nitroblue tetrazolium assay with non-denaturing polyacrylamide gel (NBT-PAGE).[13] The Amplex® Red method for detection of H_2O_2 was used as previously reported for LPMOs characterization.[14] Briefly, 100 µmol L^{-1} Amplex® Red/HRP (7 U mL^{-1}) was mixed with 50 µM ascorbic acid and the reaction was started adding 1 µM of *Cg*SOD-1. As control, the reaction above was also performed using 1 µmol L^{-1} of Cu/Zn *Bt*SOD1 (Ref_Seq NP777040) from *Bos Taurus* and purchased from Sigma-Aldrich (S9697). The change of absorbance at 560 nm was measured every 30 s for 30 min at 30 °C. The reaction was also performed in the presence of 1 mmol L^{-1} diethyldithiocarbamate (DDC) as Cu/Zn SOD inhibitor. The absorbance values of a blank reaction containing Amplex® Red and ascorbic acid or Amplex® Red, ascorbic acid and DDC were subtracted from test reactions. A hydrogen peroxide standard curve was constructed for its quantification.

Spectroscopic methods

Far-UV circular dichroism (CD) measurements were carried out using a Jasco J-810 spectropolarimeter (Jasco International Co. Ltd., Tokyo, Japan), equipped with a Peltier temperature control unit, from 190 to 260 nm in a 1 mm path length quartz cuvette according to Mandelli *et al.*.[10] For thermal stability assays, the protein sample was heated from 20 to 95 °C at a rate of 1 °C min^{-1} and Far-UV scan was acquired at 220 nm for each temperature. All the spectra were corrected by discounting the solvent contribution. The CD data are shown as mean residue ellipticity units (deg cm^2 $dmol^{-1}$). The secondary structure contents were evaluated by deconvolution of the CD spectrum using the DichroWeb K2d database.[15] The molecular modelling of *Cg*SOD-1 was determined using the online server SWISS-MODEL[16] and the crystal structure of superoxide dismutase from the silkworm *Bombyx mori* (*Bm*SOD1- PDB: 3L9Y, with 76% identity)[17] was used as a template. The final model was generated with high confidence based on the QMEAN4 score[18] of 1.045. The APBS plugin in PyMol software was used to calculate and for visualize the surface electrostatic potential at pH 6.0 ± 2 KBT/e.

The small-angle X-ray scattering (SAXS) dataset for *Cg*SOD-1 was collected on the SAXS2 beamline at the Brazilian Synchrotron Light Laboratory (Campinas, Brazil) with monochromatic X-ray λ= 1.55 Å. X-ray patterns were recorded using a two-dimensional detector (MarResearch, USA) at 1 and 5 mg/mL in 20 mM phosphate buffer (pH 6.0). The sample-to-detector distance was set to 1,000 mm to give a range of the scattering vector q from 0.015 to 0.34 $Å^{-1}$, where q is the magnitude of the q-vector defined by $q = 4\pi \sin\theta/\lambda$ (2θ is the scattering angle). The SAXS patterns were integrated using Fit2D software and the curves were scaled by the protein concentration.[19] The radius of gyration (R_g) of the molecules was determined by two independent methods, from Guinier equation[20] and by indirect Fourier transform method using Gnom package.[21] The distance distribution function p(r) was also evaluated with GNOM software, and the maximum diameter (D_{max}) was obtained. Molecular Weight was obtained using SAXSmoW webtool.[22] Dummy atom models (DAMs) were calculated from the experimental curves from *Cg*SOD-1 by *'ab initio'* procedures implemented in Dammin package.[23] Damaver was used for automated analyses and averaging of multiple reconstructions, permitting both to analyze the stability of the reconstruction convergence and to yield the most probable particle model.[24] CRYSOL 2.7 was used to generate the simulated scattering curve,[25] R_g and D_{max} from DAM and Zn/Cu-SOD from silkworm *Bombyx mori* (PDB ID 3L9Y),[17] which has the higher identity with *Cg*SOD-1 (73%). Gnom was used to obtain the distance distribution function p(r) from the crystallographic model. Supcomb[26] was used to superimpose the *Cg*SOD-1 DAM and the PDB ID 3L9Y. Figures were generated with PyMOL v 1.7.4.

Continuous wave X-band frozen solution EPR spectra of single samples of 0.25 mmol L^{-1} solution of Cu/Zn *Cg*SOD-1 without 6xHis-tag in 50 mmol L^{-1} Tris-HCl pH 7.5 and 160 K were acquired on a Bruker micro EMX spectrometer operating at ~9.30 GHz, with a modulation amplitude of 4 G, modulation frequency of 100 kHz and microwave power of 10.02 mW. Spectral simulations were carried out using EasySpin 5.2.6[27] integrated into MATLAB R2016a software. It was assumed that *g* matrix and A tensors were axially coincident. g_3 and $|A_3|$ values were determined accurately from the three absorptions at low field. Accurate determination of the $g_{1,2}$ and $|A_{1,2}|$ was not possible due to the second-order nature of the spectrum, although it was noted that satisfactory simulations could only be achieved with the set of values reported in Supplementary Table 5. Furthermore, it was noted that the simulations were improved by the addition of coupled nitrogen nuclei, although the exact value of the coupling could not be determined given the lack of well-resolved superhyperfine (SHF) coupling, therefore the values reported in Supplementary Table 4 must be considered as a mere indication of the magnitude. Simulation parameters are given in Supplementary Table 5. Raw EPR data are available on request through Research Data York (doi: 10.15124/01a0aecb-15f3-4948-a6c4-9f1d5f7ce6e9).

Immunolocalization of *Cg*SOD-1 and *Cg*EG-1

The immunolocalizations of *Cg*SOD-1 and *Cg*EG-1 were performed in the termite gut, according to Price *et al.*[28] in biological triplicates. The heterologous expression and purifications of *Cg*EG-1 were performed according to Franco Cairo *et al.*.[29] The purified *Cg*SOD-1 and *Cg*EG-1 were used to produce polyclonal antibodies in rabbits, according to standard protocols (www.rheabiotech.com.br). IgG fractions were purified from rabbit serum according to the manufacturer's instructions. Eluted antibodies were concentrated to 10 mg mL^{-1}. For the immunolocalization of the *Cg*SOD-1 and *Cg*EG-1, workers of *C. gestroi* were washed in 70% (v/v) ethanol followed by PBS and the complete guts were dissected out in PBS with PMSF (0.2 mM), EDTA (1 mmol L^{-1}) and leupeptin (20 µmol L^{-1}). After dissection, *C. gestroi* guts were transferred to a tube containing a solution of 2% (w/v) paraformaldehyde. The gut tissues were fixed for 2 h at room temperature. After guts fixation, the samples were washed several times in PBS 1X. Nonspecific antibody binding was prevented by incubating the guts for 1 h in a solution containing 4% (v/v) Triton X-100 with 2% (w/v) bovine serum albumin (BSA) in PBS. After the blocking step, the guts were incubated in the primary antibody with shaking at 4 °C for 24 h. Anti-*Cg*SOD-1 and Anti-*Cg*EG-1 were used separately at a concentration of 1:1000 in antisera buffer (0.4% (v/v) Triton X-100 with 2% (w/v) BSA in PBS). Gut tissues were then washed in PBS at 4 °C for 24 h. Labelled secondary antibody AlexaFluor 568 (Red) was incubated with each gut tissues at a concentration of 1:200 in antisera buffer at 4 °C for 18 h. The secondary antibody solution was removed, and the tissues were washed in PBS at 4 °C for 18 h.

Immunostained guts were mounted on glass slides with ProLong™ Gold Antifade Mountant with or without DAPI. Control experiments were run in parallel, which consisted of the tissue incubation with primary antibody only or the secondary fluorescence antibody only. Control experiments were set up following the same procedure, except for the appropriate antibody incubation stage was omitted. In parallel, the pre-immune rabbit serum was used as negative control in the titer assays for antibody dilutions (See supplementary material). The slides with the entire guts were observed and recorded using a stereomicroscope with Epi-Fluorescence Illuminator (Nikon's SMZ1500) with TRITC filter (red). For the closed images, the observations and recording were performed on a Leica DMI 6000 microscope at the Biological Imaging Facility (LBI) from the Brazilian Biosciences National Laboratory (LNBio).

Detection of hydrogen peroxide in *C. gestroi* guts

Workers of *C. gestroi* fed in SCB (5 insects) were washed in 70% ethanol for 20 seconds and allowed to dry on filter paper (Whatman) for 10 seconds. Afterwards, they were immersed in 50 µL of 100 mmol L^{-1} sodium acetate buffer at pH 5.5 to remove residual ethanol and subsequently immersed in 50 µL of a solution of 100 µmol L^{-1} Amplex Red with 0.2 U mL^{-1} Horseradish peroxidase (HRP) and incubated for 1 hour in the dark at room temperature. The termites were then washed five times with 200 µL of 100 mmol L^{-1} sodium acetate buffer, pH 5.5, allowed to dry on filter paper for 30 seconds on reduced light exposure, and then transferred onto a slide for microscope observation using Axio Imager 2 - Zeiss under HXP-120 Light Source (Metal Halide Lamp) at the Biological Imaging Facility (LBI) from the Brazilian Biosciences National Laboratory (LNBio), 20% of brightness with a rhodamine excitation filter (535 nm). For *C. gestroi* gut analyses, workers were washed in 70% ethanol for 20 seconds and allowed to dry in filter paper for 10 seconds. They were subsequently transferred onto a slide with 50 µL of 100 mmol L^{-1} sodium acetate buffer at pH 5.5, and the guts were extracted using forceps. On reduced light exposure, the guts were positioned on a slide and then immersed in 20 µL of Amplex Red/HRP and incubated in the dark at room temperature for 5 minutes. After incubation, the worker's guts were washed five times on another slide with 20 µL of mmol L^{-1} mM sodium phosphate buffer at pH 7.0 and transferred to a new slide for microscopy observation using Axio Imager 2 - Zeiss under HXP-120 Light Source (Metal Halide Lamp) with 20% of brightness with a rhodamine excitation filter (535 nm). The images were acquired using AxioCam ICc1 and processed using Axio Visio software 3.2. All the analyses were performed in biological triplicates.

Assay for synergy of *Cg*SOD-1 with *C. gestroi*'s glycoside hydrolases

Activity assays were performed to assess the potential enzymatic synergism between *Cg*SOD-1 and *Cg*EG-1 - GHF-9 (an endo-β-1,4-glucosidase 1 from *C. gestroi*), for glucose-based polymers saccharification, using protein and substrate concentrations, and reaction volumes previously reported in the literature.[29–35] *Cg*EG-1, *was* heterologous expressed and purified as previously described [33,29]. Reactions were performed using 100 µL of 0.5 % β-glucan (β-1,3;1-4) from Barley (low viscosity - Megazyme) or 0.5 % carboxymethylcellulose (CMC), followed by the addition of 30 or 40 µL of 100 mmol L^{-1} of Sodium Acetate buffer pH 5.5 and 10 µL of each enzyme (50 ng or 300 ng of *Cg*EG1), and different concentration of *Cg*SOD-1: 50, 100, 300 and 500 ng). The same assay was also performed using 300 ng Cu/Zn *Bt*SOD1 as control. All the reactions were performed with at least 3 biological replicates and five technical replicates at 30 °C for 30 min in PCR plates. After saccharification, 100 µL of reaction were transferred to a new plate, followed by the addition of 100 µL of 3,5-Dinitrosalicylic Acid (DNS) solution.[36] The reactions were boiled for 5 min at 99 °C, the absorbance was measured at 540 nm, and the results were presented as released reducing sugar (µmol). A glucose standard curve was constructed for glucose equivalent quantification. The degree of synergism (DS) was calculated as $abc/(a+b+c)$ as described previously,[37] where *ab* is the result in µmol of reducing sugar released by the enzymes together and *a* and *b* are the results of each enzyme alone. DS ≥ 1.1 indicates a synergism effect for the combination of the enzymes.

Activity of *Cg*SOD-1 on model polysaccharide substrate

To understand the catalytic activity of *Cg*SOD-1 on polysaccharides, we used High-Performance Anion Exchange Chromatography with Pulsed Amperometric Detector (HPAEC-PAD) as previously described in the literature[38–40] for the detection of either native or oxidized oligosaccharides, aldonic oligosaccharides or 4-ketooligosaccharides, released from the reducing or non-reducing terminals, respectively. Reactions were performed with a final concentration of 0.25% β-glucan (β-1,3;1-4) from Barley (low viscosity - Megazyme) as polysaccharide substrate, and with 20 µg of *Cg*SOD-1 without 6xHis-tag. The final volume of reactions was set at 200 µL by addition of 50 mmol L^{-1} of Sodium Acetate Buffer pH 6. Negative control reactions were performed as described above using denatured (10 min boiled and 10 min frost) *Cg*SOD-1. All reactions were performed in triplicate at 30 °C for 120 min in 2 mL tubes without agitation. Positive control reactions containing 10 ng of Celluclast 1.5L with 0.25% β-glucan (β-1,3;1-4) from Barley were incubated during 20 and 40 minutes at 30 °C in 2 mL tubes without agitation. The samples were then centrifuged for 10 minutes at 10,000 x*g* and the supernatants were transferred to conical vials. HPAEC-PAD was conducted using an ICS3000 or ICS6000 systems (Dionex-Thermo, Sunnyvale, CA, USA) equipped with a gold electrode PAD detector. Samples of 10 µL were injected onto a column system composed of a CarboPac PA1 4×250 mm analytical column and a CarboPac PA1 4×50 mm guard column at 30 °C for ICS3000 and in column system composed of a CarboPac PA1 2×250 mm analytical column and a CarboPac PA1 2×50 mm guard column at 30 °C for ICS6000. The gradient method used was a linear gradient of 100% A:0% B to 90%A:10%B for 10 min, followed by an exponential gradient to 84% A:16% B (9 minutes) and the last exponential gradient to 100% B (6 min). Eluent A was 0.1 mol L^{-1} NaOH and eluent B was 0.1 mol L^{-1} NaOH and 1 mol L^{-1} Sodium Acetate. Celloligosaccharide standards C2-C6 were used in this condition. We used the peak retention time described by Westereng *et al.*[38] to support the interpretation of the oxidized oligosaccharide structures. The same reactions described above were also performed with r Cu/Zn *Bt*SOD1 as control.

For MALDI-TOF MS analysis, the reactions were performed with 0.5% (w/v) of β-glucan or 0.5% (w/v) Avicel as the substrate with 20 µg of *Cg*SOD-1 without 6xHis-tag. The final volume of reactions was set at 200 µL by adding 20 mmol L^{-1} of Ammonium Acetate Buffer pH 6. Control reactions were performed as described above using denatured *Cg*SOD-1. All reactions were performed in triplicate at 30 °C for 24 hours in 2 mL tubes at 1000 RPM. The samples were then centrifuged for 10 minutes at 10,000 x*g* and the supernatants were transferred to new Eppendorf tubes. Afterwards, 1 µL of reaction supernatant was mixed with 2 µL of 20 mg mL^{-1} 2,5-dihydroxybenzoic acid (DHB) in 50% acetonitrile, 0.1% TFA on a SCOUT-MTP 384 target plate (Bruker). The spotted samples were then dried under 90W lamp before being analyzed by mass spectrometry on an Ultraflex III matrix-assisted laser desorption ionization and time of flight (MALDI/TOF-TOF) instrument (Bruker). Data were collected using a 2-kHz smart beam-II laser and acquired on reflector mode (mass range 800–3000 Da) for MS analysis. FlexControl and FlexAnalysis software were used for data acquisition and analysis. On average, about 10,000 shots were used to obtain high-enough resolution.

Measurements of hydroxyl radicals

The ability of *Cg*SOD-1 to produce hydroxyl radical ($^{\bullet}OH$) was monitored using 2-6-(4-hydroxy)phenoxy-3H-xanthen-3-on-9-yl-benzoic acid or hydroxyphenyl fluorescein (HPF) as a fluorescent probe.[41] As controls, the reactions were also performed in the presence of high concentrations of H_2O_2 or ascorbic acid. For these reactions, 50 ng of *Cg*SOD-1 was incubated in darkness with 30 mmol L^{-1} H_2O_2 or 10 mM Ascorbic Acid (final concentrations), and 40 µL of 50 µmol L^{-1} HPF dissolved in sodium acetate buffer (150 mmol L^{-1} pH 6.0), to reach an a final volume of 200 µL. Additional control reactions were also performed with denatured (10 min boiled and 10 min frost) *Cg*SOD-1 or 0.1 mmol mL^{-1} $CuCl_2$. As a positive control, 10 µL of 2 mmol L^{-1} $FeSO_4$ were incubated in the dark with 40 µL of 50 mmol L^{-1} HPF dissolved in sodium acetate buffer (150 mmol L^{-1} pH 6.0), plus 50 µL of 150 mmol L^{-1} Sodium Acetate buffer pH 6. For Fenton reaction initiation, 100 µL of a 2 mmol L^{-1} H_2O_2 solution were added to the assay for a total of 200 µL. All reactions were performed in triplicates in black microplates. The assays were kinetically monitored over 30 minutes at 35 °C using a plate reader fluorometer (Molecular Devices). The excitation wavelength was 488 nm, and the emission wavelength was 515 nm. Blank reactions containing HPF only or HPF in the presence of H_2O_2 or ascorbic acid were subtracted from the tested reactions and from the positive and negative controls. The results were referred to Fluorescence Increase in Reference Fluorescence Units – RFU, in which the RFU intensity from time zero for each kinetic reaction was subtracted from each measured time point.

Lignocellulose saccharification using *Cg*SOD-1 as supplement for Celluclast® and *Penicillium echinulatum* secretome

The steam-exploded sugarcane bagasse (BEX) (51.7% cellulose, 8.9% hemicellulose and 34.3% lignin)[42] was subjected to enzymatic saccharification with a commercially available enzyme preparation (Celluclast® 1.5L, Sigma - Novozymes). Reactions with 5 and 10 FPU per g^{-1} of bagasse of Celluclast alone or in combination with 2 or 20 µg of *Cg*SOD-1 as final amounts were performed with 2% (w/v) of pretreated sugarcane bagasse (30 mg) in 100 mmol L^{-1} sodium citrate buffer pH 5.5 at 30 °C and 1000 RPM. The reactions were carried out in a final volume of 1.5 mL in 2 mL Eppendorf tubes using a Thermomixer microplate incubator (Eppendorf, Germany). After incubation, the samples were centrifuged at 10,000 x*g* for 15 min (5418 Centrifuge, Eppendorf) and filtrated (Sepak C18, Waters). As negative controls denatured (10 min boiled and 10 min frost) *Cg*SOD-1 was used in all reaction lacking the active enzyme. Moreover, a control reaction containing 10 FPU Celluclast® and 20 µg of *Cg*SOD-1 was performed with 1 mmol L^{-1} diethyldithiocarbamate (DDC) as a Cu/Zn SOD inhibitor. The supplementation assays were also performed in the same condition described above using 20 µg of *Bt*SOD-1 as well as using 20 µg of *Cg*SOD-1

with and without the His-tag. The analyses were performed in triplicate. Following saccharification, the quantification of released sugars and determination of the degree of synergism (DS) were performed as described in the section above. The supplementation assays using *Cg*SOD-1 with or without the His6x-tag did not evidence differences in DS between them (Fig. S5b).

Penicillium echinulatum S01M29 was grown according to Costa *et al.* [42] and the secretome was recovered, concentrated using VivaSpin 3 kDa and washed with 100 mmol L^{-1} sodium acetate buffer pH 4.8 for the experiments. Hydrothermally pretreated sugarcane bagasse (BH) was used as substrate (61.3% cellulose, 6.9% hemicellulose and 28.8% lignin).[43] For supplementation assays, 0.33 FPU of *P. echinulatum* secretome per g^{-1} of substrate was added in 5% (m/v) of BH in 100 mmol L^{-1} sodium acetate buffer pH 4.8. Different amounts of *Cg*SOD-1 were supplemented in the reactions (0-150 µg/mL) to reach a final volume of 1.5 mL and mix at 50 °C, 1,000 rpm for 24 hours using a thermo mixer (ThermoMixer C, Eppendorf). After incubation, the samples were centrifuged at 10,000 x*g* for 15 min (5418 Centrifuge, Eppendorf) and filtrated (Sepak C18, Waters). The whole analyses were performed in biological triplicates and five technical replicates. The quantification of released sugars and determination of the degree of synergism (DS) were performed as described above.

Supporting Results

SAXS analyses

According to small angle X-ray scattering (SAXS) experiments, *Cg*SOD-1 shows high flexibility, according to Porod and Kratky analyses (Fig. S3A and S3B) [44]. The Porod constant was determined with the value of 3.1744^{-5}. Applying the Guinier approximation in the scattering curve (qR_g <1.3) [20], the obtained radius of gyration of *Cg*SOD-1 was equal to 21.1 Å (Fig. S3C). These values are in agreement with those obtained by Gnom (21.30 ± 0.03) Å [21]. The distance distribution function *p(r)* of *Cg*SOD-1 was slightly displaced to the left and fits well with a Cu/Zn SOD from *Bombyx mori* (PDB 3L9Y) [17], with D_{max} = 65 Å (Fig. S3D). Using SAXSMoW server [22], it was possible to estimate the molecular mass of *Cg*SOD-1 as being 33.2 kDa. These values indicate the predominance of dimers in solution, and the relative error was of 1.6% compared to the theoretical primary sequence of 17.9 kDa.

The SAXS *'ab initio'* model was built using the Dammin package [23]. To check the uniqueness of the model, independent simulations with different initial parameters, and without imposing symmetry, were performed. SAXS model resolution is given by the resolution of the *'ab initio'* model, and in this case is equal to 18.52 Å. Three-dimensional dummy atom model (DAM) of *Cg*SOD-1 was determined from the SAXS curves (Fig. 3E). Dammin also calculated and subtracted the Porod constant resulting in a calculated value of $3.275e^{-5}$. The experimental scattering curve overlaps well with the simulated curve of the dummy atom model in a befitting manner. The curve of simulated scattering coordinates of the dummy atom and the high-resolution models were also calculated [25] (Table S3).

Supporting Figures

A)

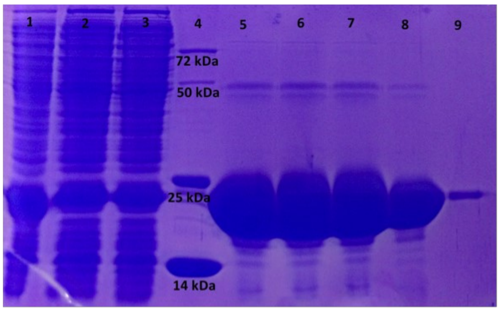

B)

Predict Protein	Best Match Organism	Contigs in *C. gestroi* database	Protein Score	Peptides Sequence	Peptide Counts	m/z (charge)
Superoxide dismutase	*Triatoma infestans*	c96668 and c98154	4980	SACGVVGIAK	2	481.2709 (2+)
				VTGEVTGLSK	22	495.7774 (2+)
				DHGGPDDEVR	52	548.7423 (2+)
				LISLTGAHNIIGR	55	682.8948 (2+)
				TLVVHADPDDLGK	34	690.3883 (2+)
				GTLFFDQENPDSAVK	64	834.4284 (2+)
				HVGDLGNIEADSSGVAK	25	834.9400 (2+)
				VDIADKLISLTGAHNIIGR	25	481.2715 (2+)
				TLVVHADPDDLGKGGHELSK	7	696.7095 (3+)
				GTLFFDQENPDSAVKVTGEVTGLSK	9	880.4706 (3+)
				DHGGPDDEVRHVGDLGNIEADSSGVAK	3	916.1204 (3+)
				GLHGFHIHEFGDNTNGCTSAGAHFNPYQK	1	1071.8490 (3+)

C) **>CgSOD-1**

MPIKAVCVLNGEVVKGTLFFDQENPDSAVKVTGEVTGLSKGLHGFHIHEFGDNTNGCTSAGAHFNPYQKDHG GPDDEVRHVGDLGNIEADSSGVAKVDIADKLISLTGAHNIIGRTLVVHADPDDLGKGGHELSKTTGNAGARSA CGVVGIAKI

Figure S1. Recombinant production of *Cg*SOD-1. The heterologous expression of *Cg*SOD-1 in *E. coli* ArcticCell Expression System. The enzyme was expressed at 12 °C, 120 rpm for 24 hours and purified throughout IMAC and Gel Filtration chromatography. **(A)** 12% SDS-PAGE analysis of *CgSOD*-1 - lane 1-3: lysate, flow through and wash fraction; lanes 5 - 8: IMAC fractions*;* lane 9: Gel filtration fraction of *Cg*SOD-1 (~ 22 kDa). **(B)** Mascot Search output with the list of peptides identified using LC-MS/MS derived from lane 5 in the SDS-PAGE. **(C)** Amino acid sequence of *Cg*SOD-1; In red are the peptides identified in LC-MS/MS.

A.

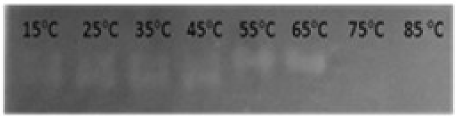

B.

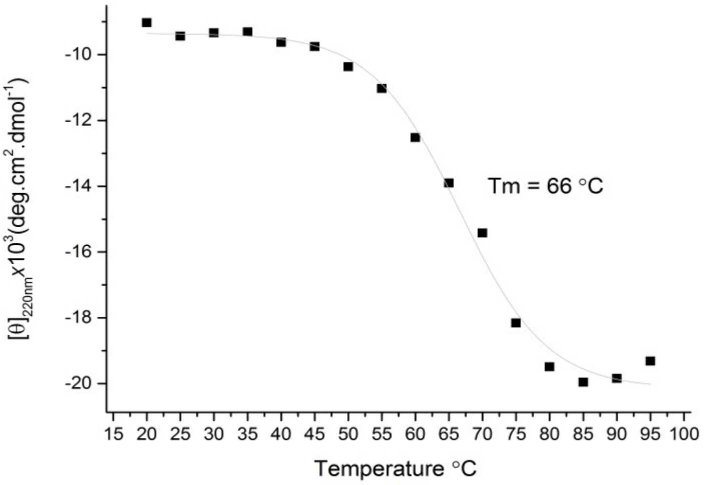

C.

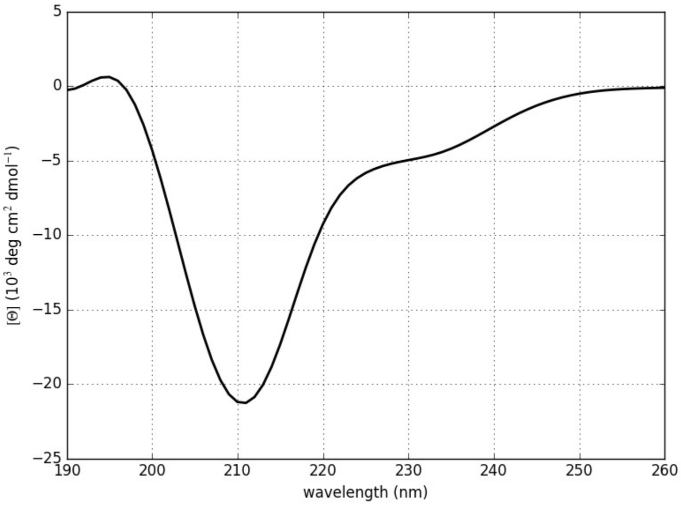

Figure S2. Thermo stability and FAR-UV Circular dichroism analysis of *Cg*SOD-1. (A) Activity of *CgSOD-1* at different temperatures (15°C to 85°C), using the riboflavin-nitroblue tetrazolium assay. White halos correspond to positive activity. **(B)** Monitoring at 220 nm the thermal denaturation of *Cg*SOD-1 from 20 to 100 °C and the calculated temperature of melting (T_m). **(C)** Far-UV CD spectrum of *Cg*SOD-1 at 20 °C. The experiments were carried out using 0.2 mg/mL of Cu/Zn loaded *Cg*SOD-1 in 20 mM sodium phosphate buffer. Deconvolution of CD data showed that the *Cg*SOD-1 secondary structure is formed by 8% α-helix, 47% β-sheet and 44% random coil at 20 °C, in agreement with the conserved family folding[35].

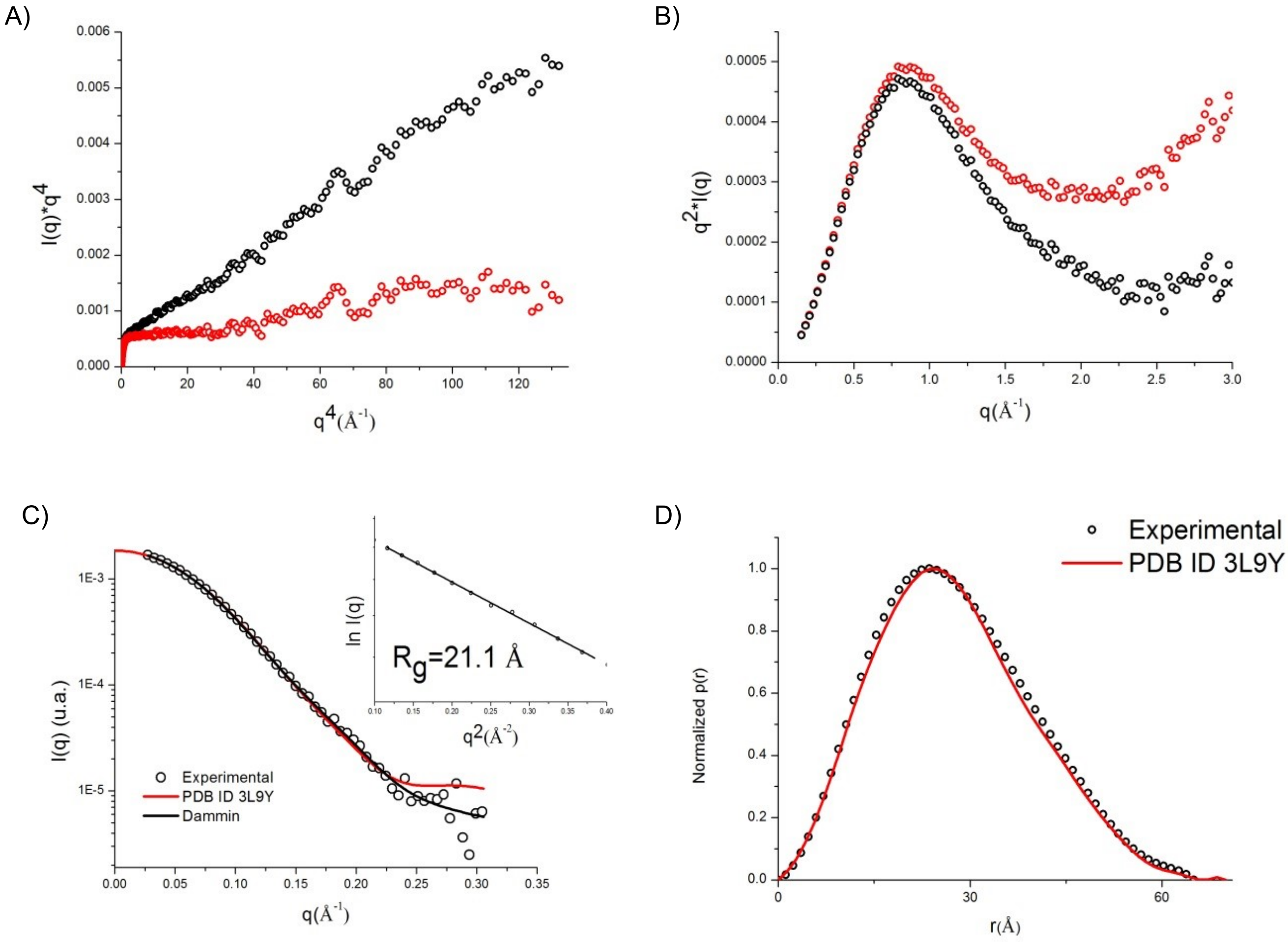

Figure S3. SAXS analysis of *CgSOD-1*. SAXS curve of *Cg*SOD-1 **(A)** Porod and **(B)** Kratky plot. Data before (red circles) and after (black circles) the subtraction of the Porod constant. **(C)** Experimental SAXS curve of *Cg*SOD-1 and fitting procedure with simulated curves. Experimental data at 5 mg/mL as open circles, PDB ID 3L9Y data as a red line, model 'ab initio' dammin data as a black line. Insert containing Guinier analysis. **(D)** Normalized p(r)s.

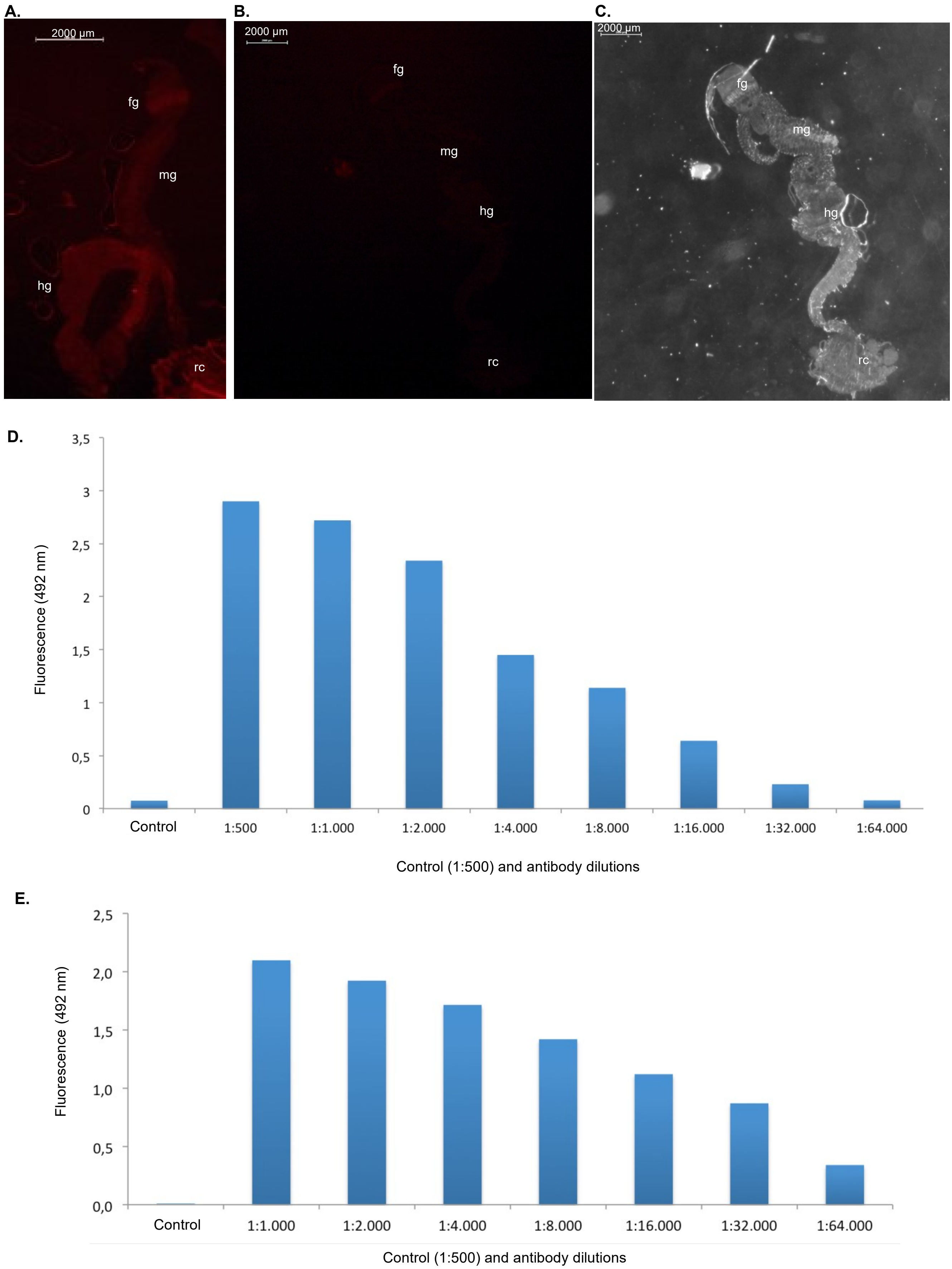
A.
2000 µm
fg
mg
hg
rc
B.
2000 µm
fg
mg
hg
rc
C.
2000 µm
fg
mg
hg
rc
D.
Fluorescence (492 nm)
3,5
3
2,5
2
1,5
1
0,5
0
Control
1:500
1:1.000
1:2.000
1:4.000
1:8.000
1:16.000
1:32.000
1:64.000
Control (1:500) and antibody dilutions
E.
Fluorescence (492 nm)
2,5
2,0
1,5
1,0
0,5
0,0
Control
1:1.000
1:2.000
1:4.000
1:8.000
1:16.000
1:32.000
1:64.000
Control (1:500) and antibody dilutions

Figure S4. Immunolocalization controls of *Cg*EG-1 and *Cg*SOD-1 in *C. gestroi* gut tissue. Gut tissues were incubated only with fluorescent secondary antibody AlexaFluor 568 for *Cg*EG-1 **(A)** and for *Cg*SOD-1 **(B)**. Phase-contrast of figure B **(C)**. Gut tissues were also incubated only with primary antibody where no fluorescence was detected (data not shown). The slides were mounted using ProLong™ Antifade Reagents for Fixed Cells (without DAPI) and observed on a stereomicroscope with Epi-Fluorescence Illuminator (Nikon's SMZ1500) with TRITC filter (red). The images were recorded using fluorescence with rodhopsin filter (red). Gut legends: fg- foregut, mg - midgut, hg - hindgut and rc - rectum. Titer assays for testing anti-*Cg*SOD-1 **(D)** and for anti-*Cg*EG-1 **(E)** antibodies were performed to validate their specificity. The purified IgG fractions from rabbits were tested for specific binding to their targets by using standard indirect enzyme-linked immunosorbent assays (ELISAs). The results showed that the anti-CgSOD-1 and anti-*Cg*EG-1 antibodies could bind specifically with recombinant *Cg*SOD-1 and *Cg*EG-1 (at 5 µg/mL both) respectively. The assays were performed by RHEABIOTECH LTDA as described in the Materials and Methods section and the company's specifications. The control reactions were performed by using the pre-immune serum from the respective rabbit at 1:500 dilution. The blocking step was carried out in phosphate-buffered saline (PBS; 137 mM NaCl, 2.7 mM KCl, 10 mM Na_2HPO_4 and 2 mM KH_2PO_4, pH 7.5) plus 2% BSA, and the detection was carried out with the following antibodies dilutions: 1:500, 1:1,000, 1:2,000, 1:4,000, 1:8,000, 1:16,000, 1:32,000, and 1:64,000. The revelation step was performed with a secondary antibody conjugated with peroxidase, and H_2O_2/OPD was used as the chromogen substrate. The reading was carried out at TECAN 200 Pro spectrophotometer at 492 nm.

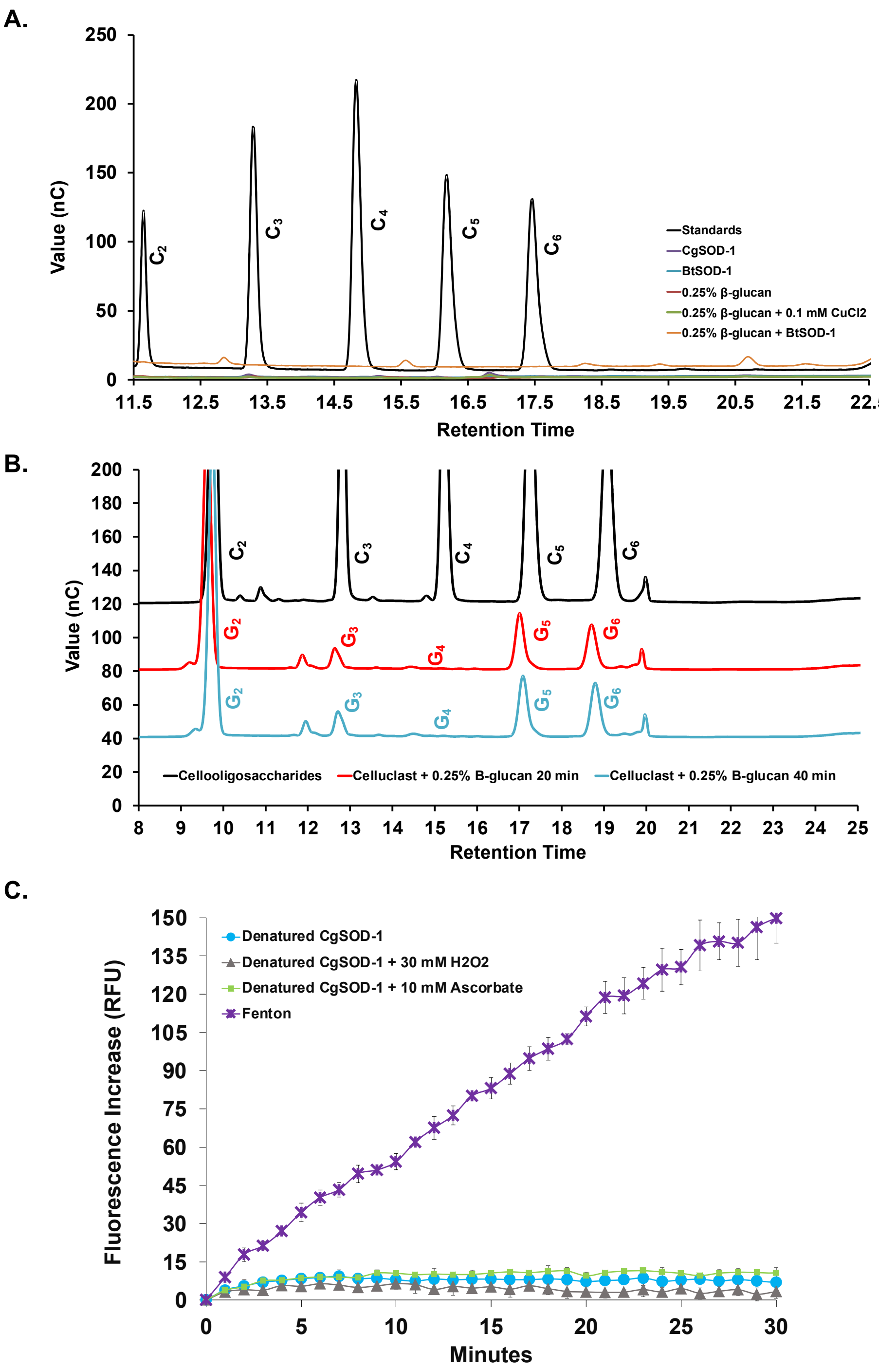

Figure S5. Reaction controls of oxidative cleavage of β-glucan by *Cg*SOD-1 or *Bt*SOD-1 and the generation of hydroxyl radicals. (A) HPAEC (ICS3000) chromatograms from negative reaction controls containing: celloligosaccharide standards, 0.25% β-glucan, 0.25% β-glucan + 0.1 mM $CuCl_2$, only *Cg*SOD-1 or *Bt*SOD-1and 0.25% β-glucan after incubation with *Bt*SOD-1. **(B)** HPAEC (ICS6000) chromatograms from positive reaction controls containing: 0.25% β-glucan + Cellulclast®. **(C)** The generation of hydroxyl radicals by denatured *Cg*SOD-1. Fluorescence increasing relative to the hydroxyl radical (•OH) formation detected by HPF probe at pH 6. Reactions were carried out with 50 ng of denatured *Cg*SOD-1 (10 min boiled and 10 min frost) alone or in the presence of 30 mM hydrogen peroxide or 10 mM ascorbic acid. Fenton reaction (1 mM H_2O_2 + 0.1 mM $FeSO_4$ [Fe^{2+}] - final concentrations) was used as positive control.

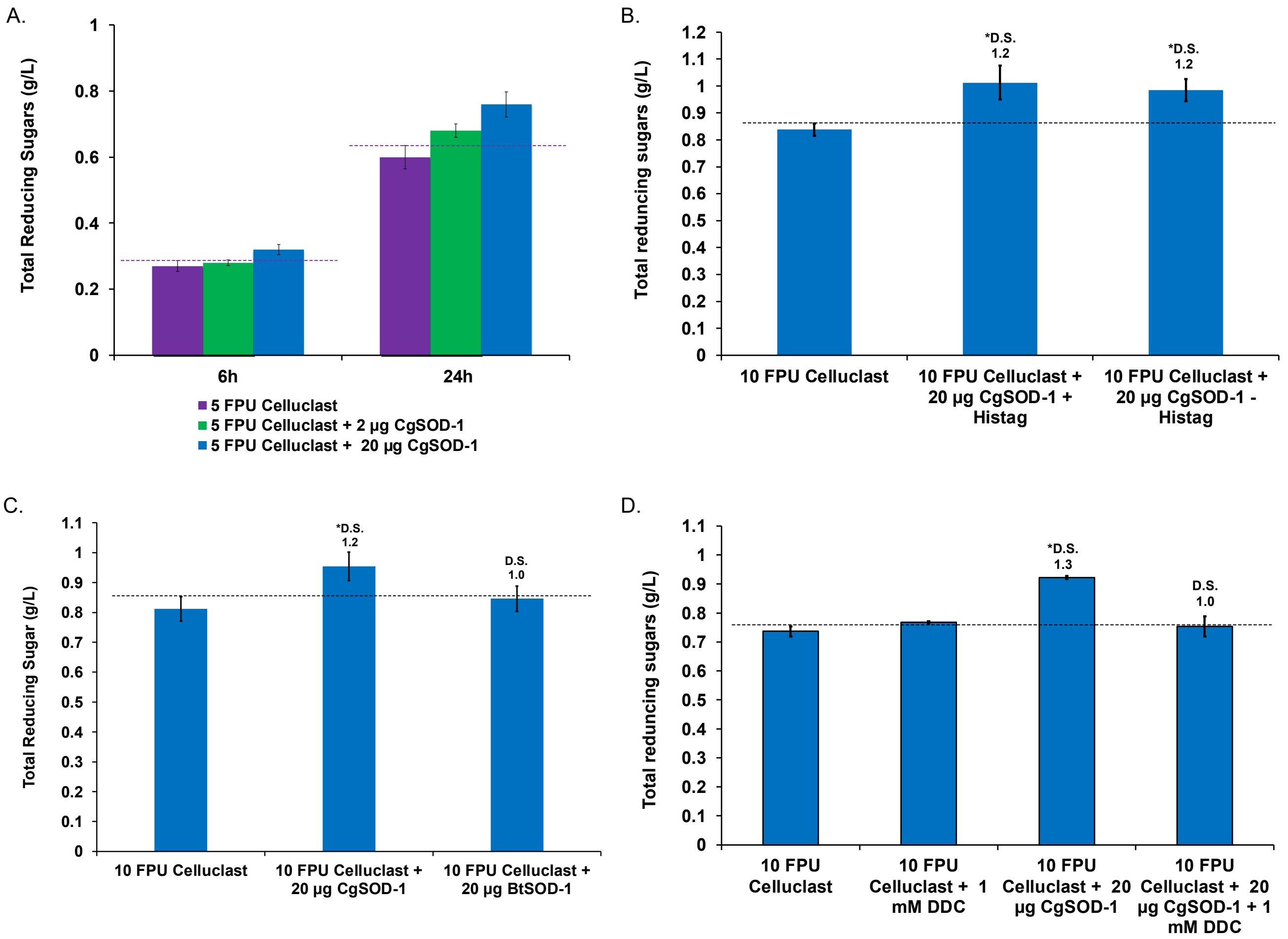

Figure S6. Control reactions of synergistic effects among superoxide dismutases and Celluclast®. (A) Supplementation of 5 FPU of ®Celluclast and two different concentrations of *Cg*SOD-1 (2 and 20 µg). The saccharification was performed at 30 °C for 24 hours with 2% of steam-exploded sugarcane bagasse (BEX) in 1.5 mL of total reaction volume. After the assays, the total reducing sugars were measured with DNS method and the D.S. was calculated. DS ≥ 1.1 indicates a synergism effect for the combination of the enzymes. **(B)** Supplementation of Celluclast® (10 FPU) performed using 20 µg of *Cg*SOD-1 with and without 6xHis-tag. (C) Supplementation of ®Celluclast (10 FPU) performed using 20 µg of *Bt*SOD-1 or *Cg*SOD-1. **(D)** Supplementation of Celluclast® (10 FPU) was performed using 20 µg of *Cg*SOD-1 in the presence of 1 mM diethyldithiocarbamate, a specific Cu/Zn chelator used for superoxide dismutase inhibition. 1mM of DDC alone was not able to inhibit the saccharification using only Celluclast®. The saccharification assays were performed at 30 °C for 24 hours with 2% of steam-exploded sugarcane bagasse (BEX) in 1.5 mL of total reaction volume.

Supporting Tables.

Table S1. List of SOD and Actin genes identified in the genome of *C. gestroi* and their expression analysis in feeding experiment.

Contigs	Genes	Taxonomic Origin	Conserved Domain	Average RPKM in SCB	Average RPKM in PASCB	Average RPKM in DELSCB	PASCB X SCB		DELSCB X SCB		Wolf-Psort Localization
							logFC	EDGER FDR	logFC	EDGER FDR	
evm.model.scaffold57797.2	*CGSOD-1*	Insect	Sod_Cu - Pfam00080	347.3	113.7	169.3	-0.90	0.001	-0.96	0.000	extr: 31
evm.model.scaffold142449.1	*CGSOD-2*	Insect	Sod_Cu - Pfam00080	41.8	12.6	32.1	-1.13	0.019	-0.34	0.565	n.d.
evm.model.scaffold22018.1	*CGSOD-3*	Insect	Sod_Cu - Pfam00080	135.7	82.5	103.7	-0.36	0.511	-0.49	0.014	plas: 11, extr: 11
evm.model.scaffold22547.2	*CGSOD-4*	Insect	Sod_Cu - Pfam00080	49.3	21.6	43.0	-0.10	1.000	0.19	0.970	n.d.
evm.model.scaffold22547.1	*CGSOD-5*	Insect	Sod_Cu - Pfam00080	38.4	21.6	38.8	-0.24	1.000	-0.04	1.000	extr: 26
evm.model.scaffold3342.3	*CGSOD-6*	Insect	Sod_Cu - Pfam00080	39.4	29.3	43.5	-0.24	0.993	-0.12	0.974	extr: 30
evm.model.scaffold41699.1	*CGSOD-7*	Insect	Sod_Cu - Pfam00080	64.3	34.7	37.2	-0.19	1.000	-0.63	0.000	extr: 26
evm.model.scaffold43589.2	*CGSOD-8*	Insect	Sod_Cu - Pfam00080	127.1	41.1	60.4	-1.00	0.031	-0.96	0.001	extr: 31
evm.model.scaffold8229.1	*CGSOD-9*	Insect	Sod_Cu - Pfam00080	180.1	42.7	69.5	-0.93	0.004	-0.99	0.000	n.d.
evm.model.scaffold15669.1	*CGBG-1*	Insect	GH1 - Pfam00232	1083.8	144.5	325.8	-1.7	2E-11	-1.3549	6.5E-08	extr: 22
evm.model.scaffold15714.1	*CGBG-2*	Insect	GH1 - Pfam00233	906.3	188.8	385.5	-1.6	1E-08	-1.2302	1.3E-07	extr: 28
evm.model.scaffold1416.1	*CGEG-1*	Insect	GH9 - Pfam00759	25883.1	4837.0	8488.9	-1.4	1E-05	-1.1708	3.2E-06	extr: 29
gene_2909+|9051|9314	*CGEG-2*	Insect	GH9 - Pfam00760	11262.0	2603.9	4669.0	-1.3	4E-04	-1.1696	1.6E-06	extr: 26
evm.model.scaffold138433.1	*CGACTN-1*	Insect	Actin - Pfam00022	8035.0	4682.0	3451.4	-0.47	0.296	-0.56	0.007	n.d.
evm.model.scaffold143434.1	*CGACTN-2*	Insect	Actin - Pfam00023	91.4	60.3	38.7	-0.03	1.000	-0.12	0.891	n.d.
evm.model.scaffold14599.2	*CGACTN-3*	Insect	Actin - Pfam00024	46.6	22.9	21.2	-0.43	0.961	-0.50	0.352	n.d.

Table S2. List of SOD and Actin proteins identified in the proteome of *C. gestroi* and their expression during the feeding experiment.

Proteins	Conserved Domains	Average and Normalized Spectrum Counts		Fold Change	Fisher's Exact (p-value)	Secretion Signal
		Raw SCB	PASCB			
***Cg*SOD-1**	Sod_Cu - Pfam00080	31 (±5.7)	14.67 (±2.6)	2.1	0.0036	Yes
***Cg*SOD-2**	Sod_Cu - Pfam00080	0.33 (±0.2)	0.33 (±0.2)	0	0.62	No
***Cg*SOD-9**	Sod_Cu - Pfam00080	4 (±0.8)	3 (±0.8)	1.3	0.28	No
***Cg*BG-1**	GH1 - Pfam00232	8.33 (± 6.3)	19.1 (±6.2)	1.8	0.14	Yes
***Cg*BG-2**	GH1 - Pfam00233	2.0 (±1.7)	3.3 (±0.5)	2.2	0.32	Yes
***Cg*EG-1**	GH9 - Pfam00759	36.5 (±10.1)	26.6 (±5.0)	0.7	0.0001	Yes
***Cg*EG-2**	GH9 - Pfam00760	42.5 (±5.0)	33.3 (±11.0)	0.8	0.0002	Yes
***Cg*ACTN-1**	Actin - Pfam00022	63 (±11.3)	63.4 (±1.2)	1	0.36	No
***Cg*ACTN-6**	Actin - Pfam00022	17.2 (±5.4)	16.9 (±1.5)	1	0.27	No

Table S3. SAXS parameters from *Cg*SOD-1 in aqueous solution.

Parameters	5 mg	Modelo 'ab initio' Dammin	Crysol
R_g(Å)	21.1(Guinier)<br>21.30(Gnom)	-	20.09
D_{max}(Å)	65	-	70.88
MW SaxsmoW (kDa)	31.1	-	-
Resolution(Å)	18.52	18.52	-
χ	-	1.387	2.480

Table S4. EPR simulation parameters for *Cg*SOD-1.

Parameters		Values
	g_1	2.020
g values	g_2	2.105
	g_3	2.268
	$|A_1|$	160
A_{Cu} (MHz)	$|A_2|$	70
	$|A_3|$	410
SHF A_N (isotropic) (MHz)		36, 38, 40
A_{cu} strains (MHz)		60, 85, 60
Line widths		0.6, 0.6
Frequency (GHz)		9.29069

References

1 N. K. Hapukotuwa and J. K. Grace, *Insects*, 2011, **2**, 499–508.
2 S. C. Rabelo, C. E. Vaz Rossell, G. J. de Moraes Rocha and G. Zacchi, *Biotechnol. Prog.*, 2012, **28**, 1207–1217.
3 S. M. de Vasconcelos, A. M. P. Santos, G. J. M. Rocha and A. M. Souto-Maior, *Bioresour. Technol.*, 2013, **135**, 46–52.
4 G. Siqueira, A. Várnai, A. Ferraz and A. M. F. Milagres, *Appl. Energy*, 2013, **102**, 399–402.
5 J. P. L. Franco Cairo, M. F. Carazzolle, F. C. Leonardo, L. S. Mofatto, L. B. Brenelli, T. A. Gonçalves, C. A. Uchima, R. R. Domingues, T. M. Alvarez, R. Tramontina, R. O. Vidal, F. F. Costa, A. M. Costa-Leonardo, P. Leme, A. F, G. A. G. Pereira and F. M. Squina, *Front. Microbiol.*, , DOI:10.3389/fmicb.2016.01518.
6 M. D. Robinson, D. J. McCarthy and G. K. Smyth, *Bioinformatics*, 2010, **26**, 139–140.
7 P. Horton, K. J. Park, T. Obayashi, N. Fujita, H. Harada, C. J. Adams-Collier and K. Nakai, *Nucleic Acids Res*, 2007, **35**, W585-7.
8 A. Keller, A. I. Nesvizhskii, E. Kolker and R. Aebersold, *Anal Chem*, 2002, **74**, 5383–5392.
9 E. Gasteiger, C. Hoogland, A. Gattiker, S. Duvaud, MarcR. Wilkins, RonD. Appel and A. Bairoch, in *The Proteomics Protocols Handbook*, ed. JohnM. Walker, Humana Press, 2005, pp. 571–607.
10 F. Mandelli, J. P. L. Franco Cairo, a. P. S. Citadini, F. Büchli, T. M. Alvarez, R. J. Oliveira, V. B. P. Leite, a. F. Paes Leme, a. Z. Mercadante and F. M. Squina, *Lett. Appl. Microbiol.*, 2013, **57**, 40–46.
11 K. Helsens, E. Timmerman, J. Vandekerckhove, K. Gevaert and L. Martens, *Mol. Cell. Proteomics MCP*, 2008, **7**, 2364–72.
12 S. Marklund and G. Marklund, *Eur. J. Biochem.*, 1974, **47**, 469–474.
13 A. Boonmee, C. Srisomsap, A. Karnchanatat and P. Sangvanich, *J. Med. Plants Res.*, 2011, **5**, 5208–5215.
14 R. Kittl, D. Kracher, D. Burgstaller, D. Haltrich and R. Ludwig, *Biotechnol. Biofuels*, 2012, **5**, 79.
15 L. Whitmore and B. A. Wallace, *Nucleic Acids Res.*, 2004, **32**, W668–W673.
16 M. Biasini, S. Bienert, A. Waterhouse, K. Arnold, G. Studer, T. Schmidt, F. Kiefer, T. G. Cassarino, M. Bertoni, L. Bordoli and T. Schwede, *Nucleic Acids Res.*, 2014, **42**, W252–W258.
17 N.-N. Zhang, Y.-X. He, W.-F. Li, Y.-B. Teng, J. Yu, Y. Chen and C.-Z. Zhou, *Proteins*, 2010, **78**, 1999–2004.
18 P. Benkert, M. Künzli and T. Schwede, *Nucleic Acids Res.*, 2009, **37**, W510–W514.
19 A. P. Hammersley, *FIT2D: An Introduction and Overview*, 1997.
20 A. Guimer and G. Fournet, *J Wiley Sons N. Y.*
21 A. V. Semenyuk and D. I. Svergun, *J. Appl. Crystallogr.*, 1991, **24**, 537–540.
22 H. Fischer, M. de Oliveira Neto, H. B. Napolitano, I. Polikarpov and A. F. Craievich, *J. Appl. Crystallogr.*, 2010, **43**, 101–109.
23 D. I. Svergun, *Biophys. J.*, 1999, **76**, 2879–2886.
24 V. V. Volkov and D. I. Svergun, *J. Appl. Crystallogr.*, 2003, **36**, 860–864.
25 D. Svergun, C. Barberato and M. H. J. Koch, *J. Appl. Crystallogr.*, 1995, **28**, 768–773.
26 M. B. Kozin and D. I. Svergun, *J. Appl. Crystallogr.*, 2001, **34**, 33–41.
27 S. Stoll and A. Schweiger, *J. Magn. Reson.*, 2006, **178**, 42–55.
28 D. R. G. Price, J. Du, a Dinsmore and J. a Gatehouse, *Insect Mol. Biol.*, 2004, **13**, 469–80.
29 J. P. L. Franco Cairo, L. C. Oliveira, C. a. Uchima, T. M. Alvarez, A. P. D. S. Citadini, J. Cota, F. C. Leonardo, A. M. Costa-Leonardo, M. F. Carazzolle, F. F. Costa, G. a G. Pereira and F. M. Squina, *Insect Biochem. Mol. Biol.*, 2013, **43**, 970–981.
30 F. Sabbadin, G. R. Hemsworth, L. Ciano, B. Henrissat, P. Dupree, T. Tryfona, R. D. S. Marques, S. T. Sweeney, K. Besser, L. Elias, G. Pesante, Y. Li, A. A. Dowle, R. Bates, L. D. Gomez, R. Simister, G. J. Davies, P. H. Walton, N. C. Bruce and S. J. McQueen-Mason, *Nat. Commun.*, 2018, **9**, 756.
31 F. Sabbadin, S. Urresti, B. Henrissat, A. O. Avrova, L. R. J. Welsh, P. J. Lindley, M. Csukai, J. N. Squires, P. H. Walton, G. J. Davies, N. C. Bruce, S. C. Whisson and S. J. McQueen-Mason, *Science*, 2021, **373**, 774–779.
32 K. Besser, G. P. Malyon, W. S. Eborall, G. P. da Cunha, J. G. Filgueiras, A. Dowle, L. C. Garcia, S. J. Page, R. Dupree, M. Kern, L. D. Gomez, Y. Li, L. Elias, F. Sabbadin, S. E. Mohamad, G. Pesante, C. Steele-King, E. R. de Azevedo, I. Polikarpov, P. Dupree, S. M. Cragg, N. C. Bruce and S. J. McQueen-Mason, *Nat. Commun.*, 2018, **9**, 5125.
33 R. Tramontina, J. P. L. Franco Cairo, M. V. Liberato, F. Mandelli, A. Sousa, S. Santos, S. C. Rabelo, B. Campos, J. Ienczak, R. Ruller, A. R. L. Damásio and F. M. Squina, *Biotechnol. Biofuels*, 2017, **10**, 4.
34 A. Sethi, J. M. Slack, E. S. Kovaleva, G. W. Buchman and M. E. Scharf, *Insect Biochem Mol Biol*, 2013, **43**, 91–101.
35 M. E. Scharf, Z. J. Karl, A. Sethi and D. G. Boucias, *PLoS One*, 2011, **6**, e21709.
36 G. L. Miller, *Anal. Chem.*, 1959, **31**, 426–428.
37 J. Woodward, *Bioresour. Technol.*, 1991, **36**, 67–75.
38 B. Westereng, J. W. Agger, S. J. Horn, G. Vaaje-Kolstad, F. L. Aachmann, Y. H. Stenstrøm and V. G. H. Eijsink, *J. Chromatogr. A*, 2013, **1271**, 144–152.
39 D. Cannella, K. B. Möllers, N.-U. Frigaard, P. E. Jensen, M. J. Bjerrum, K. S. Johansen and C. Felby, *Nat. Commun.*, 2016, **7**, 11134.
40 B. Westereng, J. S. M. Loose, G. Vaaje-Kolstad, F. L. Aachmann, M. Sørlie and V. G. H. Eijsink, in *Cellulases: Methods and Protocols*, ed. M. Lübeck, Springer, New York, NY, 2018, pp. 219–246.
41 K. -i. Setsukinai, Y. Urano, K. Kakinuma, H. J. Majima and T. Nagano, *J. Biol. Chem.*, 2003, **278**, 3170–3175.
42 P. dos Santos Costa, F. Büchli, D. Robl, P. da S. Delabona, S. C. Rabelo and J. G. da C. Pradella, *J. Ind. Microbiol. Biotechnol.*, 2016, **43**, 627–639.
43 D. A. Ribeiro, J. Cota, T. M. Alvarez, F. Brüchli, J. Bragato, B. M. P. Pereira, B. A. Pauletti, G. Jackson, M. T. B. Pimenta, M. T. Murakami, M. Camassola, R. Ruller, A. J. P. Dillon, J. G. C. Pradella, A. F. Paes Leme and F. M. Squina, *PloS One*, 2012, **7**, e50571–e50571.
44 R. P. Rambo and J. A. Tainer, *Biopolymers*, 2011, **95**, 559–571.
